# Newly Developed CK1-Specific Inhibitors Show Specifically Stronger Effects on CK1 Mutants and Colon Cancer Cell Lines

**DOI:** 10.3390/ijms20246184

**Published:** 2019-12-07

**Authors:** Congxing Liu, Lydia Witt, Chiara Ianes, Joachim Bischof, Marie-Thérèse Bammert, Joana Baier, Stefan Kirschner, Doris Henne-Bruns, Pengfei Xu, Marko Kornmann, Christian Peifer, Uwe Knippschild

**Affiliations:** 1 Department of General and Visceral Surgery, Ulm University Hospital, Albert-Einstein-Allee 23, 89081 Ulm, Germany; congxing.liu@uni-ulm.de (C.L.); chiara.ianes@uni-ulm.de (C.I.); joachim.bischof@uniklinik-ulm.de (J.B.); marie-therese.bammert@hochschule-bc.de (M.-T.B.); doris.henne-bruns@uniklinik-ulm.de (D.H.-B.); pengfei.xu@uniklinik-ulm.de (P.X.); marko.kornmann@uniklinik-ulm.de (M.K.); 2 Institute of Pharmacy, Christian-Albrechts-University of Kiel, Gutenbergstraße 76, 24118 Kiel, Germany; lwitt@pharmazie.uni-kiel.de (L.W.); jbaier@pharmazie.uni-kiel.de (J.B.); skirschner@pharmazie.uni-kiel.de (S.K.)

**Keywords:** CK1, small molecule inhibitors, Michaelis–Menten kinetics, casein kinase 1, kinase mutant

## Abstract

Protein kinases of the CK1 family can be involved in numerous physiological and pathophysiological processes. Dysregulated expression and/or activity as well as mutation of CK1 isoforms have previously been linked to tumorigenesis. Among all neoplastic diseases, colon and rectal cancer (CRC) represent the fourth leading cause of cancer related deaths. Since mutations in CK1δ previously found in CRC patients exhibited increased oncogenic features, inhibition of CK1δ is supposed to have promising therapeutic potential for tumors, which present overexpression or mutations of this CK1 isoform. Therefore, it is important to develop new small molecule inhibitors exhibiting higher affinity toward CK1δ mutants. In the present study, we first characterized the kinetic properties of CK1δ mutants, which were detected in different tumor entities. Subsequently, we characterized the ability of several newly developed IWP-based inhibitors to inhibit wild type and CK1δ mutants and we furthermore analyzed their effects on growth inhibition of various cultured colon cancer cell lines. Our results indicate, that these compounds represent a promising base for the development of novel CRC therapy concepts.

## 1. Introduction

CK1δ, a member of the CK1 family (formerly named casein kinase 1 family), is involved in regulation of different cellular processes—among them cell cycle, mitosis, DNA damage signaling, and developmental pathways. Dysregulation of CK1δ expression and/or activity has been observed in different types of disorders, including cancer. So far, different CK1δ mutations have been already identified and seem to have a higher oncogenic potential in comparison to CK1δ wild type [1,2]. Recently, the CK1δ^T67S^ mutant has been identified in colon and rectal cancer (CRC) and this mutant showed increased in vitro kinetics and is associated with increased cell proliferation and tumor growth in both cell culture and in a subcutaneous tumor xenotransplantation mouse model [2]. Another CK1δ mutant, CK1δ^R324H^, has been observed in adenomas of intestinal mucosa and seems to have carcinogenic potential, which may influence tumorigenic development [3]. Moreover, some mutations in CK1δ can also affect its kinase activity, such as the S97C amino acid exchange, which seems to play a key role for ATP-binding and subsequent phosphorylation events [1]. In addition, dimerization of overexpressed kinase-dead mutants with wild type CK1δ can also negatively affect the kinase activity of (endogenous) CK1δ [4]. So far, different additional CK1δ mutations have been already identified in different cancer studies and have been collected by the cBioPortal for Cancer Genomics database [5,6]. Nevertheless, no information about their influence on kinase activity and oncogenic potential has been known so far.

Due to the oncogenic potential of CK1 isoforms, numerous small molecule inhibitors have been developed during recent years with the aim to achieve beneficial effects in the treatment of different tumor entities. For the CK1-specific inhibitor IC261, therapeutic potential could be observed in xenotransplantation models for pancreatic cancer and neuroblastoma tumors [7,8]. However, off-target effects due to direct binding to microtubules and due to blockage of voltage-gated sodium channels can also be triggered by IC261 [9,10]. In cell culture and mouse models for chronic lymphocytic leukemia (CLL), treatment with the CK1δ- and ε-specific inhibitor PF-670462 resulted in a longer overall survival [11]. Inhibitor PF-4800567 showed increased selectivity toward CK1ε compared to CK1δ and induced remarkable anti-proliferative effects in different cell lines [12]. Furthermore, benzimidazole-based CK1 isoform-specific inhibitors like SR-3029, Bischof-5, Richter-2, and IWP-based compounds were demonstrated to inhibit viability and/or proliferation of tumor cell lines of different origin—among them also cell lines derived from colon and rectum tissue [13,14,15,16,17]. Although cure rates of colon and rectal cancer patients are continuously increasing for early and locally advanced stages (UICC I-III) [18], the prognosis is still poor for patients displaying distant metastases in liver or lung. At present, a five-year survival rate of 12% for stage IV disease has been reported for the US [18]. In order to further improve outcome, treatment of stage IV disease has to be optimized probably by further individualization according to the respective disturbances found [19,20].

This study focused on the characterization of CK1δ mutants, which were identified in various aggressive tumor types, among them CRC, and to characterize their effects on kinase activity and enzyme kinetics. This study also focused on characterization of the sensitivity of hyperactive mutants toward small molecule inhibitors, compared to CK1δ wild type, which may broaden the therapeutic window for potential future clinical applications. Moreover, we analyzed the ability of these compounds to inhibit the growth of established primary and metastasis-derived colon cancer cell lines.

## 2. Results

### 2.1. Kinetic Analysis of CK1δ Mutants Identified in Different Tumor Entities

**Figure 1:**
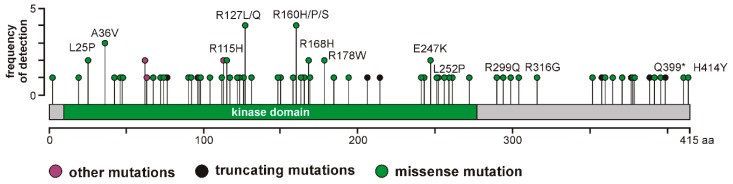
Mutations in *CSNK1D* identified in different types of cancer. According to the database cBioPortal for Cancer Genomics, 123 mutations have been reported in a curated set of 159 non-redundant studies, including 42,199 samples [5,6]. Positions of the respective mutations in the CK1δ protein and their frequency of detection are indicated. Missense mutations are displayed in green, truncating mutations in black, and other types of mutations (e.g., protein fusion mutations) in purple. Highlighted mutations were selected for further analysis. Abbreviations: A, alanine; aa, amino acids, E, glutamic acid; G, glycine; H, histidine; I, isoleucine; K, lysine; L, leucine; M, methionine; P, proline; Q, glutamine; R, arginine; S, serine; V, valine; W, tryptophan; Y, tyrosine; *, stop codon.

Various mutations of *CSNK1D* have been identified and reported for different types of cancer. The database cBioPortal for Cancer Genomics lists a total of 123 mutations identified for *CSNK1D* in a curated set of 159 non-redundant studies (Figure 1 and Table 1) [5,6]. Based on their high recurrence or identification in aggressive tumor entities, among them colon and rectal adenocarcinoma, the mutations highlighted in Figure 1 were selected for further characterization in biochemical and cell biological analysis.

**Figure 2:**
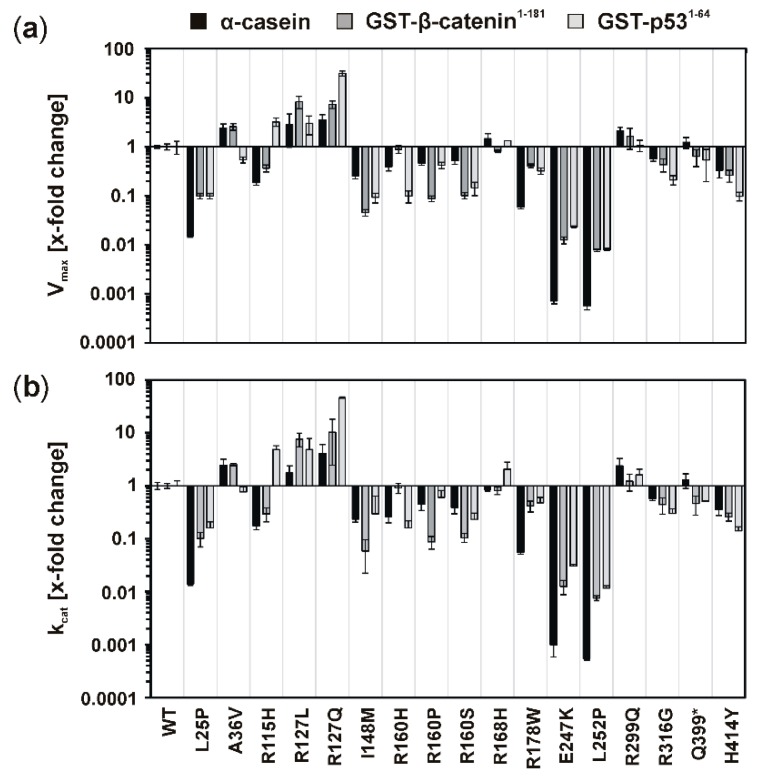
Kinetic parameters V_max_ and k_cat_ of CK1δ wild type and mutants with different substrates. Michaelis–Menten kinetics have been analyzed for CK1δ wild type and mutants using either α-casein, GST-β-catenin^1−181^, or GST-p53^1−64^ as substrate. The kinetic parameters V_max_ (**a**) and k_cat_ (**b**) were normalized toward the respective parameters determined for CK1δ wild type. Data is presented as mean values and standard deviation (SD) for experiments performed in triplicate. Abbreviations: A, alanine; E, glutamic acid; G, glycine; GST, glutathione S-transferase; H, histidine; I, isoleucine; K, lysine; k_cat_, turnover number; L, leucine; M, methionine; P, proline; Q, glutamine; R, arginine; S, serine; V, valine; V_max_, maximum enzyme reaction velocity; W, tryptophan; WT, wild type; Y, tyrosine; *, stop codon.

So far, no information has been available concerning the effects and the role of these mutations on CK1δ activity and function. Therefore, the corresponding mutations were inserted in a prokaryotic expression vector enabling for expression of glutathione S-transferase (GST)-CK1δ fusion proteins in *Escherichia coli*. Subsequently, kinetic analysis was performed in order to analyze potential alterations in CK1δ activity. Using substrates α-casein, GST-β-catenin^1−181^, and GST-p53^1−64^ Michaelis–Menten kinetics were performed for GST-CK1δ wild type and mutated kinases. The kinetic parameters maximal enzyme reaction velocity (V_max_) and turnover number (k_cat_) are displayed in Figure 2, while additional parameters, such as K_M_ and k_cat_/K_M_, are summarized in Supplementary Table S1.

Basically, kinetic data revealed substrate-specific differences, which were most significant for mutant R115H, displaying remarkably decreased kinase activity compared to wild type CK1δ for substrates α-casein and GST-β-catenin^1−181^ (5.4- and 2.7-fold lower, respectively) while kinase activity was remarkably increased for substrate GST-p53^1−64^ (3.2-fold increased)_._ For almost half of the tested mutants (eight out of 17), at least ten-fold lower kinase activity compared to wild type CK1δ could be observed (Figure 2). Interestingly, except for R168H, CK1δ mutations between amino acids Ile-148 and Leu-252 presented lower V_max_ and k_cat_ compared to the wild type. Mutants E247K and L252P even showed almost no residual kinase activity and presented very low V_max_ and k_cat_ values of 0.0007 and 0.0005-fold less, respectively, compared to the wild type (Table S1). In contrast, hyperactive mutants were also identified, presenting significantly higher values for V_max_ and elevated k_cat_ compared to the wild type. These include mutants A36V, R115H, R127L, R127Q, R168H, R299Q, and Q399 *.

### 2.2. Hyperactive CK1δ Mutants Are More Sensitive to Different CK1-Specific Inhibitors

As already described in previous studies, mutations in the coding sequence for CK1δ cannot only influence the activity of the enzyme but also the binding and efficacy of small molecule inhibitors [2,21]. Therefore, the sensitivity of CK1δ hyperactive mutants toward different CK1-specific inhibitors was analyzed and residual kinase activities upon inhibitor treatment are summarized in Table 2. Interestingly, all tested inhibitors (Bischof-5 [14], Richter-2 [17], IWP-2, and IWP-4 [15,22]) showed significantly stronger inhibitory effects on CK1δ^R127Q^ compared to CK1δ^WT^, leading to a residual activity between 19% and 37%. In addition, kinase activity of CK1δ^R168H^ was also influenced stronger by the benzimidazole-based compounds Bischof-5 and Richter-2. Remarkably stronger inhibition than for CK1δ^WT^ could also be observed for CK1δ^R115H^ applying Bischof-5 as well as for CK1δ^R299Q^ when using IWP-2. Being most sensitive to benzimidazole-based inhibitor compounds and IWPs, mutants CK1δ^R115H^, CK1δ^R127Q^, and CK1δ^R168H^ were selected for subsequent analysis.

### 2.3. Modeling of ATP Binding to CK1δ Mutants

**Figure 3:**
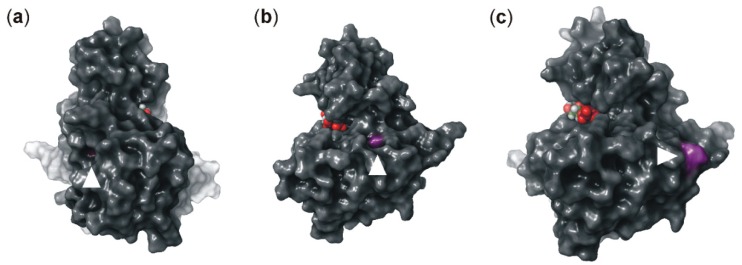
Molecular modeling of MgATP in mutated CK1δ. (**a**) CK1δ^R115H^, (**b**) CK1δ^R127Q^, and (**c**) CK1δ^R168H^ with MgATP (red/white atoms) bound in the ATP-binding pocket. The mutated residues are highlighted in purple and are indicated with white arrowheads. Induced fit calculations were performed based on a homology model of 4twc [14] on 1csn [23] CK1δ crystal structures (obtained from protein data bank (PDB) [24]) in order to realize the adjustment of the P-loop.

Modeling of MgATP in the mutated CK1δ structures (R115H, R127Q, and R168H) was performed to evaluate differences in binding of MgATP compared to the original binding pose within the ATP active site of wild type CK1δ. In fact, all tested mutations seemed to have no effect on binding of MgATP itself, as they were not situated in close proximity to the ATP-binding pocket. In Figure 3, the respective mutations are highlighted with regard to the apo structure. In line with this notion, performing SiteMap calculations at these mutated structures indicated that all three mutations were located at a potential protein substrate-binding region.

The significantly increased inhibition of CK1δ^R127Q^ by compounds Bischof-5, Richter-2, IWP-2, and IWP-4 suggests an effect of the amino acid exchange toward the active site such as a spatial adjustment of the ATP-binding pocket. However, and in contrast to the wet lab data, our modeling data does not reflect this effect regarding inhibitor binding poses.

In the case of CK1δ^R168H^, an impact toward the active site is implied as this mutation is located within the activation loop. However, in order to define these influences on inhibitor binding in more detail, further molecular dynamic simulations would be required. In order to illustrate the situation, the result of inhibitor docking of Bischof-5 in CK1δ^R168H^ is shown in Figure S1.

### 2.4. Synthesis of New IWP-Based Inhibitor Compounds

**Scheme 1:**
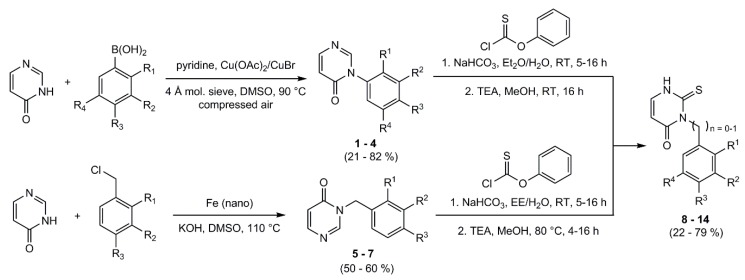
Scheme of synthesis of compounds **1**–**7** and **8**–**14**. Compounds **1**–**7**: **1** (R1−R4 = H); **2** (R1 = O CH_3_, R2−R3 = H, R4 = O CH_3_); **3** (R1 = O CH_3_, R2 = H, R3 = O CH_3_, R4 = H); **4** (R1 = H, R2 = O CH_3_, R3 = H, R4 = O CH_3_); **5** (R1–R2 = H, R3 = O CH_3_); **6** (R1–R2 = H, R3 = CF_3_); **7** (R1 = F, R2-R3 = H). Compounds **8**–**14**: **8** (n = 0, R1–R4 = H); **9** (n = 0, R1 = O CH_3_, R2-R3 = H, R4 = O CH_3_); **10** (n = 0, R1 = O CH_3_, R2 = H, R3 = O CH_3_, R4 = H); **11** (n = 0, R1 = H, R2 = O CH_3_, R3 = H, R4 = O CH_3_); **12** (n = 1, R1–R2 = H, R3 = O CH_3_); **13** (n = 1, R1–R2 = H, R3 = CF_3_); **14** (n = 1, R1 = F, R2-R3 = H).

Previously, more profound inhibitory effects on a CK1δ gatekeeper mutant (CK1δ^M82F^) in comparison to CK1δ wild type have been determined for several IWP compounds [15]. In order to identify IWP-based inhibitor compounds stronger inhibiting CK1δ mutants than CK1δ^WT^, new compounds were designed based on the IWP backbone structure. Chan–Lam coupling of compounds **1**−**4** was performed based on the literature (Scheme 1) [25]. Instead of using an oxygen filled balloon, compressed air was passed through the solvent. Compound **1** was already described by Abd El Kader et al. [26]. Benzyl compounds **5**−**7** were synthesized based on Roopan et al. using nano iron under basic conditions to provide selective N-substitution [27]. Introduction of the thioxo group (compounds **8**−**14**) followed the procedure of Xu and Yadan using phenyl thionochloroformate [28]. Compound **8** as well has been described in the literature [29].

**Scheme 2:**
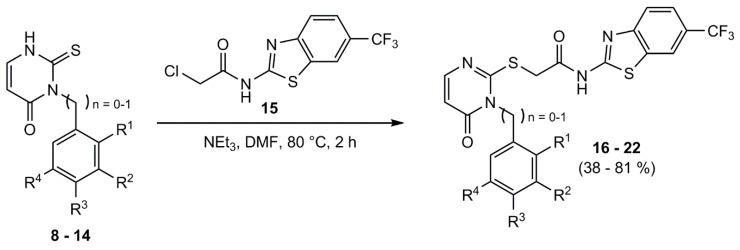
Scheme of synthesis of compounds **16**–**22**. Coupling of compounds **8**–**14** with the benzothiazole–linker **15** was performed according to the literature [30]. Compounds: **16** (n = 0, R1–R4 = H); **17** (n = 0, R1 = O CH_3_, R2-R3 = H, R4 = O CH_3_); **18** (n = 0, R1 = O CH_3_, R2 = H, R3 = O CH_3_, R4 = H); **19** (n = 0, R1 = H, R2 = O CH_3_, R3 = H, R4 = O CH_3_); **20** (n = 1, R1–R2 = H, R3 = O CH_3_); **21** (n = 1, R1–R2 = H, R3 = CF_3_); **22** (n = 1, R1 = F, R2–R3 = H).

The synthesis of the benzothiazole–linker was already described by García-Reyes and Witt et al. [15]. The coupling-conditions of compounds **8**−**14** with the benzothiazole–linker **15** were the same as for previous IWP derivatives (Scheme 2) [15,30].

### 2.5. Characterization of Newly Developed IWP-Derivatives

**Figure 4:**
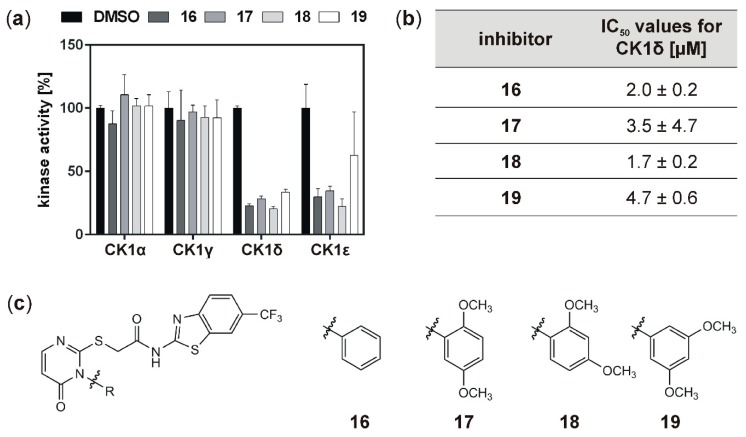
Compounds **16**, **17**, **18**, and **19** significantly inhibit kinase activity of CK1δ and ε. (**a**) Kinase activity of CK1α, γ, δ, and ε was analyzed in presence of 10 µM of newly designed IWP-derivatives **16**, **17**, **18**, and **19**. Results are presented as mean values of experiments performed in triplicate. Error bars indicate the standard error. (**b**) In vitro IC_50_ values were determined for CK1δ and a dilution series of compounds **16**, **17**, **18**, or **19**. IC_50_ values were calculated using GraphPad Prism 6. Results are presented as mean values ± standard error of experiments performed in triplicate. (**c**) Structures of inhibitor compounds **16**, **17**, **18**, and **19**. Abbreviations: DMSO, dimethyl sulfoxide; IC_50_; 50% inhibitory concentration; µM, micromolar.

Newly developed IWP-derivatives were first tested in vitro for their inhibitory potency and selectivity among wild type CK1 isoforms α, γ, δ, and ε (Figure 4a). All tested compounds (**16**, **17**, **18**, and **19**) strongly inhibited kinase activity of CK1δ and ε, while activity of CK1α and γ was not affected. Subsequently, IC_50_ values for CK1δ were determined for all new compounds in order to characterize the inhibitory effects on CK1δ in more detail. Among this set of inhibitors, **18** showed the strongest effect and the lowest IC_50_ value (1.7 μM). Also for **16**, an IC_50_ value within a similar range could be determined (2 μM) (Figure 4b).

**Figure 5:**
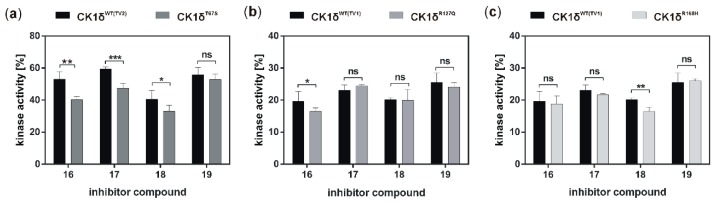
CK1δ^WT^ and selected hyperactive CK1δ mutants are inhibited by newly developed CK1-specific inhibitors. Inhibition of CK1δ^WT^ and mutants CK1δ^T67S^ (**a**), CK1δ^R127Q^ (**b**), or CK1δ^R168H^ (**c**) by IWP-derivatives **16**, **17**, **18**, and **19** at a concentration of 10 µM has been determined by in vitro kinase assays. Please note that the *Y*-axis in (**a**) is set to a maximum of 80%, while in (**b**,**c**) it is set to 40%. Residual kinase activity has been normalized toward the respective DMSO control activity (100%). Results are presented as mean values of experiments performed in triplicate. Error bars represent the SD. Statistical analysis was done by performing two-way ANOVA using following levels of significance: * *p* ≤ 0.05; ** *p* ≤ 0.01; *** *p* ≤ 0.001; ns, not significant. Abbreviations: H, histidine; Q, glutamine; R, arginine; S, serine; T, threonine; TV, transcription variant; WT, wild type.

In addition to wild type CK1δ the new compounds were also tested for inhibition of mutants CK1δ^R127Q^ and CK1δ^T168H^. Moreover, the previously published hyperactive mutant CK1δ^T67S^ was also included. Interestingly, inhibitors **16**, **17**, and **18** inhibit the hyperactive mutant CK1δ^T67S^ slightly stronger when compared to wild type CK1δ (Figure 5a). Kinase activity of CK1δ^R127Q^ was also significantly stronger inhibited by **16** (Figure 5b), while strongest effects on CK1δ^R168H^ activity could be observed for compound **18** (Figure 5c).

### 2.6. IWP-Derivative 18 Selectively Inhibits Cellular CK1δ and Shows Stronger Effects on HeLa Cells Expressing Hyperactive CK1δ Mutants

**Figure 6:**
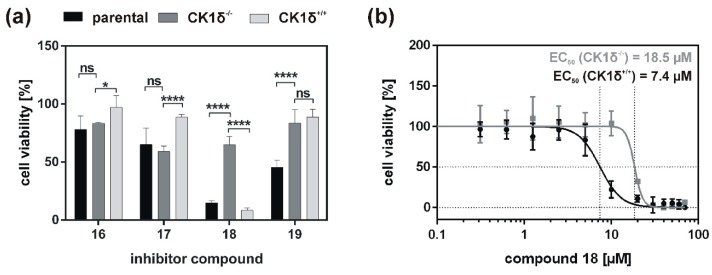
Compounds **18** and **19** show CK1δ-specific effects on cell viability. (**a**) MTT viability assays have been performed by treating parental HeLa cells, HeLa CK1δ^−/−^, or HeLa CK1δ^+/+^ cells with 25 μM of compounds **16**, **17**, **18**, **19**, or DMSO as control for 48 h. Cell viability of treated cells has been normalized toward the viability of DMSO-treated cells. Results are presented as mean values of experiments performed in triplicate. Error bars represent the SD. Statistical analysis was done by performing two-way ANOVA using following levels of significance: * *p* ≤ 0.05; **** *p* ≤ 0.0001; ns, not significant. (**b**) EC_50_ values were determined by treating HeLa CK1δ^−/−^ and HeLa CK1δ^+/+^ cells with compound **18** in a concentration range between 0.313 and 70 µM for 48 h. Cell viability of treated cells has been normalized toward the viability of DMSO-treated cells. Results are presented as mean values of experiments performed in triplicate. Error bars represent the SD. EC_50_ values have been calculated using GraphPad Prism 6. Abbreviations: EC_50_, 50% effective concentration; µM, micromolar.

Following biochemical characterization, the effects of newly designed IWP-based compounds on cell viability were analyzed for cells being deficient for CK1δ (HeLa CK1δ^−/−^) and cells expressing CK1δ (either parental HeLa or HeLa CK1δ^−/−^ being stably transfected with CK1δ, termed HeLa CK1δ^+/+^) (Figure 6a). Interestingly, compound **18** and to a lesser extent also compound **19** seemed to have a CK1δ-dependent effect on cell viability. In fact, cell viability of the CK1δ-expressing cells (HeLa parental or CK1δ^+/+^) was more reduced by treatment with compound **18** or **19** compared to CK1δ-deficient cells (HeLa CK1δ^−/−^) (Figure 6a). Moreover, 50% effective concentration (EC_50_) values were determined for HeLa CK1δ^+/+^ and CK1δ^−/−^ cells, demonstrating that viability of cells expressing CK1δ was significantly stronger affected by treatment with **18** (Figure 6b). In contrast, effects on cell viability observed for parental cells treated with **16** or **17** were less pronounced than for compounds **18** and **19**, and the effects on cell viability were not reduced for CK1δ^−/−^ cells compared to parental and/or CK1δ^+/+^ cells (Figure 6a).

**Figure 7:**
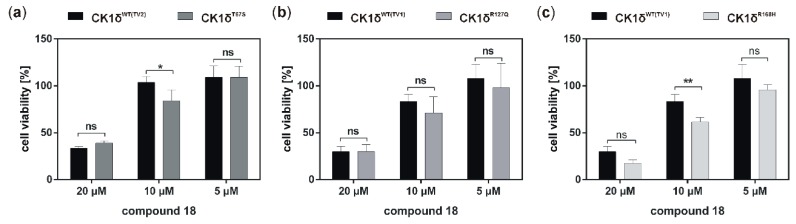
Compound **18** shows stronger effects on cell viability of HeLa cells expressing hyperactive CK1δ mutants. Cell viability of HeLa CK1δ^−/−^ cells expressing CK1δ^WT^, CK1δ^T67S^ (**a**), CK1δ^R127Q^ (**b**), or CK1δ^R168H^ (**c**) has been determined by MTT viability assay after treatment with **18** (20 μM, 10 μM, or 5 μM) or DMSO as control for 48 h. Cell viability of treated cells has been normalized toward the viability of DMSO-treated cells. Results are presented as mean values of experiments performed in triplicate. Error bars represent the SD. Statistical analysis was done by performing two-way ANOVA using following levels of significance: * *p* ≤ 0.05; ** *p* ≤ 0.01; ns, not significant. Abbreviations: H, histidine; Q, glutamine; R, arginine; S, serine; T, threonine; TV, transcription variant; WT, wild type.

The effects of compound **18** on cell viability were also tested on HeLa CK1δ^−/−^ cells expressing the selected hyperactive CK1δ mutants CK1δ^T67S^ (Figure 7a), CK1δ^R127Q^ (Figure 7b), or CK1δ^R168H^ (Figure 7c). Interestingly, 10 μM of **18** resulted in stronger effects on cell viability of CK1δ^T67S^-, CK1δ^R127Q^-, and CK1δ^R168H^-expressing HeLa cells than on CK1δ^WT^-expressing cells, although the difference was not determined to be statistically significant in the case of CK1δ^R127Q^ (Figure 7).

### 2.7. A Second Set of IWP-Derivatives Shows Improved Inhibition of CK1δ In Vitro and Cell Line-Specific Effects on Colon Cancer Cell Lines

The tested IWP-derivatives, **16**, **17**, **18**, and **19**, demonstrated remarkable inhibition of CK1 isoforms δ and ε in vitro and especially for compounds **18** and **19** significant effects on the viability of CK1δ mutant-expressing cells could be observed. Therefore, in order to obtain additional compounds with improved inhibitory potential, a second set of IWP-derived compounds was developed and initially tested in our in vitro screening procedure. In contrast to the phenyl-substituted compounds of the first set, compounds of the second set carried a benzyl-substituted pyrimidinone.

**Figure 8:**
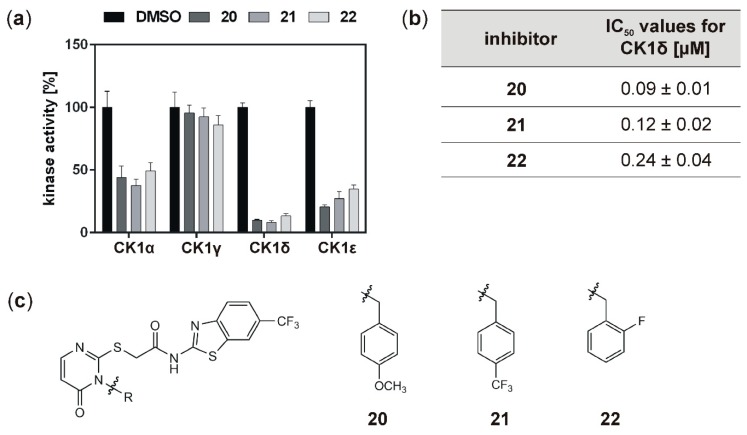
Compounds **20**, **21**, and **22** significantly inhibit kinase activity of CK1δ and ε. (**a**) Kinase activity of CK1α, γ, δ, and ε was analyzed in presence of 10 µM of newly designed IWP-derivatives **20**, **21**, and **22**. Results are presented as mean values of experiments performed in triplicate. Error bars indicate the standard error. (**b**) In vitro IC_50_ values were determined for CK1δ and a dilution series of compounds **20**, **21**, and **22**. IC_50_ values were calculated using GraphPad Prism 6. Results are presented as mean values ± standard error of experiments performed in triplicate. (**c**) Structures of inhibitor compounds **20**, **21**, and **22**. Abbreviations: DMSO, dimethyl sulfoxide; IC_50_; 50% inhibitory concentration; µM, micromolar.

All tested compounds of this second set (**20**, **21**, and **22**) strongly inhibited kinase activity of CK1δ and ε. While activity of CK1α was also reduced by at least 50% by all tested compounds, kinase activity of CK1γ was not affected (Figure 8a). Also for the second set of inhibitor compounds, the IC_50_ values for CK1δ were determined. Among this set of inhibitors, significantly lower IC_50_ values could be determined in comparison to the first set. The strongest effects and the lowest IC_50_ values of 0.086 and 0.120 µM could be determined for compounds **20** and **21**, respectively (Figure 8b).

**Figure 9:**
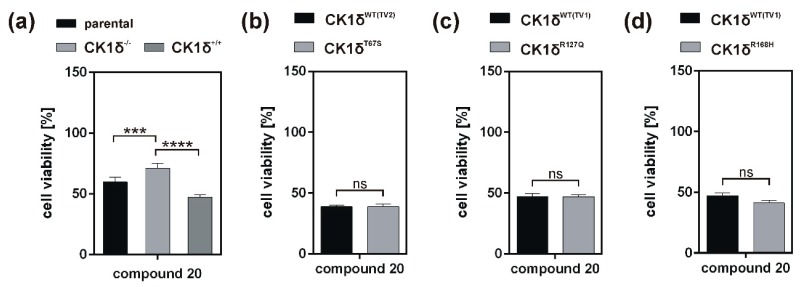
Effects mediated by compound **20** are CK1δ-specific but not mutant-specific. (**a**) MTT viability assays have been performed by treating parental HeLa cells, HeLa CK1δ^−/−^, or HeLa CK1δ^+/+^ cells with 5 μM of compound **20** or DMSO as control for 48 h. Cell viability of treated cells has been normalized toward the viability of DMSO-treated cells. Results are presented as mean values of experiments performed in triplicate. Error bars represent the SD. (**b–d**) Cell viability of HeLa CK1δ^−/−^ cells expressing CK1δ^WT^, CK1δ^T67S^ (**b**), CK1δ^R127Q^ (**c**), or CK1δ^R168H^ (**d**) has been determined by MTT viability assay after treatment with compound **20** (5 μM) or DMSO as control for 48 h. Cell viability of treated cells has been normalized toward the viability of DMSO-treated cells. Results are presented as mean values of experiments performed in triplicate. Error bars represent the SD. Statistical analysis was done by performing two-way ANOVA using following levels of significance: *** *p* ≤ 0.001; **** *p* ≤ 0.0001; ns, not significant. Abbreviations: H, histidine; Q, glutamine; R, arginine; S, serine; T, threonine; TV, transcription variant; WT, wild type.

Because compound **20** showed most remarkable inhibition of CK1δ *in vitro*, this compound was also tested for its effects on parental and CK1δ-deficient (CK1δ^−/−^) HeLa cells as well as on CK1δ-deficient HeLa cells being stably transfected with CK1δ (HeLa CK1δ^+/+^) in order to demonstrate CK1δ-selective effects. Although cell viability of HeLa CK1δ^−/−^ was significantly less affected compared to parental cells or HeLa CK1δ^+/+^ cells, observed effects of compound **20** were not as selective for CK1δ as previously determined for compound **18** (and compound **19**) (Figure 9a). Furthermore, testing of compound **20** on HeLa CK1δ^−/−^ cells stably expressing CK1δ mutants T67S, R127Q, or R168H revealed no mutant-specific effects (Figure 9b−d).

**Figure 10:**
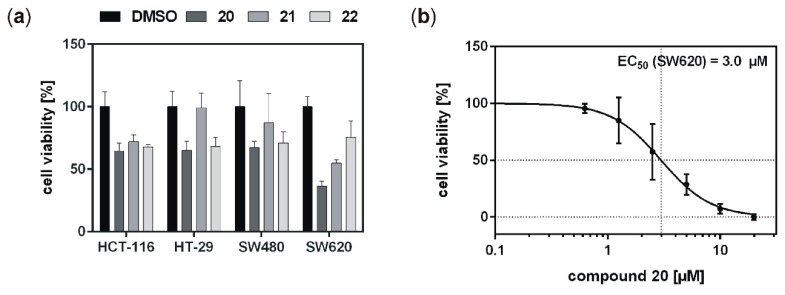
Inhibitory effects of compounds **20**, **21**, and **22** on cell viability of established colon cancer cell lines. (**a**) Inhibitors were used at a concentration of 10 µM to treat HCT-116, HT-29, SW480, and SW620 for 48 h in an initial screening. Cell viability of treated cells has been normalized toward the viability of DMSO-treated cells. Results are presented as mean values of experiments performed in triplicate. Error bars represent the SD. (**b**) EC_50_ value was determined by treating SW620 cells with compound **20** in a concentration range between 0.625 and 20 µM for 48 h. Cell viability of treated cells has been normalized toward the viability of DMSO-treated cells. Results are presented as mean values of experiments performed in triplicate. Error bars represent the SD. EC_50_ values have been calculated using GraphPad Prism 6. Abbreviations: DMSO, dimethyl sulfoxide; EC_50_, 50% effective concentration; µM, micromolar.

With regard to the potential future use of CK1 isoform-specific inhibitors in therapeutic strategies for cancer treatment, the influence of compounds **20**, **21**, and **22** on cell viability of various established colon cancer cell lines was tested by performing MTT viability assays. In an initial screening using all inhibitor compounds at a concentration of 10 µM, most of the inhibitors of the second set only showed weak effects. For cell line HCT-116, all compounds showed a reduction of cell viability by 30% to 40% (Figure 10a). For cell lines, HT-29 and SW480 inhibitors **20** and **22** also reduced cell viability by approximately 35%, whereas treatment with **21** only resulted in minor changes of viability. Strongest effects among all colon cancer cell lines could be observed for SW620. Treatment with **20** and **21** resulted in a decrease of about 60% and 50%, respectively, while a reduction of only 25% was obtained for **22** (Figure 10a).

For a more detailed characterization, the two best inhibitors per cell line were analyzed for dose-dependent effects using different concentrations ranging from 0.625 to 20 µM (Figure S2). For HCT-116 cells, treatment with inhibitor **20** as well as **22** resulted in a reduction of cell viability by approximately 30%. This could already be observed for low inhibitor concentrations of 0.625 or 1.25 µM, respectively. However, this effect is only very slightly extended by increasing inhibitor concentration and, even at a concentration of 10 µM, only 45% of viability reduction could be determined for both compounds. For HT-29 and SW480 cells, the observed effects were even less remarkable. In addition, effects of **21** on SW620 were only visible at the highest concentrations tested, whereas clear dose-dependent effects could be observed for the treatment of SW620 cells with inhibitor **20**. Obtained data even allowed for the determination of an EC_50_ value of 3 µM for compound **20** and cell line SW620 (Figure 10b and Figure S2).

## 3. Discussion

Due to the involvement of CK1 isoforms in numerous cellular processes, dysregulation of CK1 can be linked to the development of different cancers as well as to neurodegenerative diseases. Apart from an increase of expression levels, elevated CK1δ kinase activity can also be caused by mutations occurring in the genomic sequence coding for CK1δ. In order to pharmacologically control CK1δ activity, several small molecule inhibitors are already available. However, CK1 isoform-specificity and pharmacokinetic parameters for most of these inhibitors are not satisfactory and new inhibitor compounds with improved properties are required. Furthermore, specific targeting of CK1δ mutants occurring in certain tumor entities would provide a broad therapeutic window for the treatment of the respective tumor without inducing severe off-target effects in healthy tissue. In order to deal with these issues, in the present study we (i) characterized enzyme kinetic properties of CK1δ mutants identified in various cancer entities including the colon and rectum and (ii) we characterized the CK1δ wild type- and mutant-specific inhibitory potential of a small set of CK1δ-specific small molecule inhibitors in vitro and on four colon cancer cell lines.

So far, only few CK1δ mutants have been described in the literature, which could be linked to the pathogenesis of certain diseases. For instance, mutation CK1δ^S97C^ has been associated with the development of breast cancer and ductal carcinoma [1]. Mutation CK1δ^R324H^ has been linked to increased oncogenic potential and was detected in a patient with large and multiple colonic polyps. In an experimental setting, CK1δ^R324H^ has been shown to potently transform RKO colon cancer cells [3]. Although both mutants are not associated with an immediate increase of kinase activity, at least for CK1δ^R324H^, a mechanism has been detected leading to Wnt/β-catenin-independent morphogenetic movements in early *Xenopus* development [3]. Furthermore, a hyperactive mutant (CK1δ^T67S^) has been found in colon and rectal cancer and expression of CK1δ^T67S^ resulted in higher oncogenic potential in cell culture experiments as well as in a mouse xenotransplantation model [2].

In addition, 123 mutations affecting the *CSNK1D* gene are currently listed in the database cBioPortal for Cancer Genomics [5,6]. However, the effects of these mutants on CK1δ kinase activity and their oncogenic potential has not been analyzed so far. Therefore, we selected a panel of CK1δ mutations (L25P, A36V, R115H, R127L, R127Q, I148M, R160H, R160P, R160S, R168H, R178W, E247K, L252P, R299Q, R316G, Q399*, and H414Y) to subsequently perform analysis of enzyme kinetic parameters.

Elevated kinase activity could be determined for mutants GST-CK1δ^R127L^ and GST-CK1δ^R127Q^, which can be explained by changes in the electrical charge of the protein resulting from the exchange of the positively charged amino acid arginine to hydrophobic leucine or polar, but uncharged, glutamine. These changes in protein charge might result in conformational changes affecting kinase activity. Moreover, the catalytically essential residue Arg-128 is located in direct proximity to the mutated position 127, and the entire sequence between amino acids 128–133 belongs to the catalytic loop of CK1δ. Therefore, mutations within this sequence or in close proximity to it could be linked to alteration of kinase activity.

Remarkably decreased kinase activity could be observed for GST-CK1δ mutants carrying mutations I148M, R160H, R160P, R160S, R168H, R178W, E247K, or L252P. Interestingly, for mutants CK1δ^E257K^ and CK1δ^L252P^, nearly no residual kinase activity could be determined in enzyme kinetic analysis. Therefore, both mutants can be considered kinase-dead mutants. So far, also mutant CK1δ^K38M^, bearing an amino acid change at residue Lys-38, which is involved in binding of ATP, has been described as kinase-dead mutant of CK1δ [31,32].

Mutations located in the C-terminus of CK1δ (ranging from amino acid 296 to 415 for human CK1δ transcription variant 1 [33]) resulted in different effects on phosphorylation of α-casein in kinetic analysis. While increased activity was determined for GST-CK1δ^R299Q^ and GST-CK1δ^Q399*^, decreased activity was observed for GST-CK1δ^R316G^ and GST-CK1δ^H414Y^. In general, several C-terminal residues of CK1δ are involved in regulation of kinase activity, mostly by phosphorylation events mediated by intramolecular autophosphorylation or by phosphorylation by upstream kinases [21,34,35,36]. Consequently, if this domain is affected by conformational changes due to amino acid exchanges also regulation of kinase activity might be affected.

In general, mutations in the CK1δ protein might not only affect kinase activity, but can also result in different binding of small molecule inhibitors. This has previously been shown for the hyperactive mutant CK1δ^T67S^ as well as for different phosphorylation-site mutants [2,21]. Considering a potential use in therapeutic strategies for personalized medicine, stronger binding of inhibitor compounds to hyperactive CK1δ mutants could be of outstanding benefit. By selectively inhibiting CK1δ mutants expressed in tumor tissue, a wider therapeutic window is opened and side effects on tissues expressing wild type CK1δ can be limited or even avoided. In order to identify differences in sensitivity toward inhibition by specific small molecule inhibitors, certain inhibitor compounds were tested for their effects on CK1δ mutants. Compared to wild type CK1δ, the hyperactive GST-CK1δ^R127Q^ mutant could be stronger inhibited by all selected inhibitor compounds. Inhibitor-specific effects determined for incubation of GST-CK1δ^R127L^ and GST-CK1δ^R299Q^ with IWP-2 were also stronger than for wild type CK1δ and similar significant observations could as well be made for inhibition of GST-CK1δ^A36V^ and GST-CK1δ^R115H^ with Bischof-5. Mutant GST-CK1δ^R168H^ was significantly stronger inhibited by both Bischof-5 and Richter-2 when being compared to inhibitory effects observed on wild type CK1δ. In contrast, mutations in CK1δ can also lead to reduced inhibition by CK1δ-specific inhibitors. This could be mainly observed for Richter-2 and GST-CK1δ mutants A36V, R115H, and R127L, as well as for IWP-4 and mutants R299Q and Q399*.

Expression of hyperactive CK1δ mutants has been shown to be associated with increased oncogenic potential, while decreased activity of CK1δ results in reduced oncogenic functions [2,4]. Consequently, by treating tumor cells expressing hyperactive CK1δ mutants with CK1δ-specific inhibitors, beneficial effects could be obtained. With the purpose to screen for these beneficial effects, CK1δ mutant-expressing cells were treated with CK1δ-specific inhibitors. For being analyzed in this experimental approach, three CK1δ mutants were selected: mutant CK1δ^R127Q^ presented the highest activity among all tested mutants, and mutant CK1δ^R168H^ represented a unique hyperactive mutation located between Ile-148 and Leu-252. In addition, the previously described mutant CK1δ^T67S^, being characterized by increased kinase activity and high oncogenic potential, was tested in the selected cell culture model.

In order to identify inhibitors selectively targeting wild type and/or mutant CK1 isoforms, two sets of newly developed inhibitor compounds were tested within the present study. These compounds were designed based on previously reported IWP-derivatives, which demonstrated high potency and selectivity for CK1δ as well as increased affinity to a CK1δ^M82F^ gatekeeper mutant [15]. Initial screening of the IWP-derived compounds **16**, **17**, **18**, and **19** indicated that all compounds strongly inhibit CK1δ (and ε) with IC_50_ values within the one-digit micromolar range. While, in general, inhibition of the hyperactive CK1δ mutants T67S, R127Q, and R168H was within the same range as observed for wild type CK1δ, for few of the tested inhibitors slightly stronger inhibition of the mutants could be detected.

Analysis of inhibitory effects of the newly designed inhibitors on cultured cells was performed using HeLa cells. By also using HeLa cells, being deficient for CK1δ (HeLa CK1δ^−/−^), the target selectivity of the tested inhibitor compounds could be analyzed. Interestingly, treatment with compound **18** and to a lesser extent also with compound **19** (or compound **20**, which was tested in a later stage of the study) showed stronger effects on CK1δ-expressing cells than on HeLa CK1δ^−/−^ cells. We therefore conclude that at least compound **18** selectively targets CK1δ. This conclusion is supported by EC_50_ data generated for compound **18**, showing a significantly increased EC_50_ value for HeLa CK1δ^−/−^ cells. However, the effects observed for HeLa CK1δ^−/−^ cells are still obvious, which might be caused by simultaneous inhibition of other CK1 isoforms like the highly related isoform CK1ε. Furthermore, the effects obtained for compounds **16** and **17** might result from off-target effects on other CK1 isoforms or other cellular kinases in general. Significantly decreased effects on cell viability of HeLa CK1δ^+/+^ cells in comparison to parental cells could be explained by overexpression of exogenous CK1δ in HeLa CK1δ^+/+^ cells, potentially leading to highly increased intracellular target concentration.

If the hyperactive CK1δ mutants T67S, R127Q, and R168H were expressed in HeLa CK1δ^−/−^ cells, inhibitory effects mediated by compound **18** were slightly stronger. This finding underlines the marginally increased affinity of compound **18** to CK1δ mutants in comparison to wild type CK1δ. Interestingly, for all three mutant-expressing cell lines, dose-dependently different effects could be observed, thereby demonstrating the need for proper dose-response investigation.

With the aim to identify more potent CK1δ-specific inhibitors, further IWP-derived compounds of a second set were also analyzed. Compared to the first tested set, all of these compounds resulted in remarkable inhibition of CK1δ (and ε), and determined IC_50_ values for CK1δ were at least ten orders of magnitude lower than for compounds **16**, **17**, **18**, or **19**. In comparison to the originally tested IWP-compounds (IWP-2 and IWP-4; [15]), inhibitors **20**, **21**, and **22** could be significantly improved concerning their inhibitory effects on CK1δ. Because highly potent inhibitors of CK1 isoforms could be used for the treatment of proliferative diseases, the highly potent inhibitors **20**, **21**, and **22** were also tested for their effects on established tumor cell lines. Previously, CK1-specific inhibitors already showed promising effects on colon cancer cell lines and therefore, also inhibitors **20**, **21**, and **22** were subsequently tested on colon cancer cell lines HCT-116, HT-29, SW480, and SW620. While the mutation status of CK1δ (and the highly related isoform ε) in these cell lines remains to be determined and confirmed, in a previous study, we already demonstrated robust expression of CK1δ and ε as well as CK1-specific kinase activity in SW480 and SW620 cells, while HT-29 cells showed remarkably reduced expression of CK1δ as well as reduced CK1-specific kinase activity [2]. Consequently, cell line-specific differences could be observed for the tested inhibitors in our current study. The effects obtained for the treatment of HCT-116, HT-29, and SW480 cells were quite similar, although low expression of CK1δ has previously been demonstrated for HT-29 [2]. The reduction of cell viability could therefore also be mediated via the inhibition of other CK1 isoforms or targeting of other proteins in general. Furthermore, in comparison to previously described difluoro-dioxolo-benzoimidazole-derived compounds like Richter-2 [17], but also in comparison to the original IWP-compound IWP-4 [15], the cellular effects mediated by inhibitors **20**, **21**, and **22** fell short of expectations. Inadequate ability to cross the cell membrane or low target affinity within the cell might be possible reasons for the obtained results. In contrast to **21** and **22**, at least treatment with compound **20** led to comparably strong effects on viability of SW620 colon cancer cells. Keeping in mind that cell lines SW480 and SW620 derived from primary tumor (SW480) and metastasis (SW620) of the same patient, this finding is of particular interest [37]. SW480 and SW620 represent different stages of colon cancer development, and in our investigation compound **20** showed stronger effects on the metastasis compared to the primary tumor. Survival of colon and rectal cancer patients is increasing continuously and, since the prognosis of patients is mainly determined by the occurrence of liver and lung metastases with a reported 5-year overall survival of only 12% [18], successful treatment of distant metastases is desperately warranted to further improve outcome of colon and rectal cancer. With this regard, compound **20** may be an attractive agent for systemic treatment of metastatic disease. Individual screening and molecular subtyping may help in the future to identify possible candidates for such a targeted therapy [19,20].

In order to develop novel treatment strategies, detailed characterization of CK1δ (mutant)-associated functions will enable for (i) the deepened understanding of CK1δ-driven pathogenic processes and (ii) the design of new mutant-specific inhibitors with decreased off-target effects for being used in potential future therapies for various tumor entities, including colon and rectal cancer.

## 4. Materials and Methods

### 4.1. Generation of Expression Vectors

In order to introduce point mutations in CK1δ, site-directed mutagenesis using QuikChange II Site-Directed Mutagenesis Kit (Agilent, Santa Clara, CA, USA) was performed on pGEX6-P3 or pcDNA3.1 expression vectors, respectively, coding for GST-CK1δ or His-CK1δ. Mutagenesis was performed according to the manufacturer’s instructions using the primers listed in Table S2. Generation of pcDNA3.1 expression vectors coding for His-CK1δ wild type or T67S mutant was described by Richter and colleagues [2].

### 4.2. Expression and Purification of GST Fusion Proteins

Overexpression and purification of GST-CK1δ fusion proteins, either wild type or mutant, were performed as described previously [38].

### 4.3. Kinase Assays

Each kinase assay reaction was performed in kinase buffer (25 mM Tris-HCl (pH 7.5), 10 μM ATP, 10 mM MgCl_2_, 100 μM EDTA) in presence of purified GST-CK1δ kinase as source of enzyme (either wild type or mutant), an assay-specific substrate (α-casein, GST-p53^1−64^, or GST-β-catenin^1−181^), and radiolabeled ATP (2 μCi [γ-^32^P]-ATP). For performing Michaelis–Menten analysis increasing substrate concentrations were used (α-casein: 0.39−25 μM; GST-p53^1−64^: 0.09−1.87 μM; GST-β-catenin^1−181^: 0.07−1.32 μM). For biological evaluation of small molecule inhibitors (SMIs), each inhibitor compound was either tested at 10 μM concentration for initial screening or by using a dilution series (0.005−10 µM) for determination of IC_50_ values. DMSO was used as control. Reactions were incubated for 30 min at 30 °C and reactions were stopped using 5x SDS-loading buffer (250 mM Tris-HCl [pH 6.8], 25% (*v*/*v*) β-mercaptoethanol (MSH), 50% (*v*/*v*) glycerol, 10% (*w*/*v*) SDS, 0.5% (*w*/*v*) bromphenol blue). Subsequently, samples were separated by SDS-PAGE using 12.5% acrylamide gels, stained with Coomassie, and phosphorylated substrates were visualized on X-ray film. Incorporation of radiolabeled phosphate was quantified by scintillation counting of Coomassie-stained substrate bands. Defined dilutions of the reaction master mix were counted as well in order to convert results obtained as counts per minute (CPM) to units of transferred phosphate in pmol/min. Michaelis–Menten analysis was performed using GraphPad Prism 6 (GraphPad Software, San Diego, CA, USA).

### 4.4. Synthesis

All chemical reagents were commercially available from abcr GmbH, Karlsruhe, Germany; Sigma-Aldrich Chemie GmbH, Merck Group, Munich, Germany; Merck Millipore, Darmstadt, Germany; Acros Organics, Thermo Fisher Scientific, Geel, Belgium; TCI Deutschland GmbH, Eschborn, Germany; Honeywell, Morristown, NJ, USA; Grüssing GmbH, Filsum, Germany. Purchased chemicals were used without further purification. Infrared spectra (IR) were recorded on a Shimadzu IRAffinity-1S FTIR-spectrometer (Shimadzu Deutschland GmbH, Duisburg, Germany). NMR spectra were recorded on a Bruker Avance III 300 spectrometer (Bruker Corporation, Billerica, MA, USA) (300 MHz 1H frequency), (75 MHz 13C NMR frequency) and a Bruker Avance III 400 spectrometer (Bruker Corporation, Billerica, MA, USA) (400 MHz 1H frequency), (100 MHz 13C NMR frequency). Chemical shifts were reported in ppm, multiplicity, and coupling constant J (Hz). Spectra were referenced to internal DMSO-d6. Whenever appropriate, signal assignments were deduced from COSY, and CH correlation NMR experiments. LC-MS were recorded on a Bruker Esquire LC ion trap mass spectrometer spectrometer (ESI Bruker Corporation, Billerica, MA, USA). High resolution MS was performed on a Thermo Fischer Q Exactive PlusMS Hybrid Quadrupol-Orbitrap ESI-mass spectrometer (Thermo Fisher Scientific, Waltham, MA, USA). Column chromatography was performed using a LaFlash system (VWR) with Macherey–Nagel silica gel 60 (63−200 μm) for pre-columns and pre-packed Interchim PuriFlash-30SIHP silica gel columns (30 μM, 40 g) using mixtures of petroleum ether (PE) and ethyl acetate (EA). HPLC analysis was performed on a Hewlett-Packard 1050 Series using a STAGROMA YMC-C-18 column. All test compounds were proven by HPLC to have ≥97% purity. Where necessary, reactions were carried out in a nitrogen atmosphere using dry solvents.

### 4.5. General Procedure for the Synthesis of the Phenyl Pyrimidinone-Derivatives

Molecular sieve (4 Å) was dried in vacuo. 4-(3*H*)-pyrimidinone, the appropriate phenylboronic acid, and a copper catalyst were dissolved in DMSO and pyridine was added. The reaction mixture was heated to 90 °C for 4 h and compressed air was added through a small tube into the reaction mixture. After cooling, the molecular sieve and the catalyst were filtered off; the filtrate was then quenched with water and extracted with ethyl acetate. The organic phase was washed with water and brine, dried over sodium sulfate, and the solvent was removed under reduced pressure. If necessary, the product was purified by column flash-chromatography on silica gel.

3-Phenylpyrimidin-4(3*H*)-one (**1**): 3-Phenylpyrimidin-4(3*H*)-one (**1**) was obtained from 4(3*H*)-pyrimidinone (193 mg, 2.01 mmol), phenylboronic acid (487 mg, 3.99 mmol), copper(II)-acetate (72.0 mg, 0.40 mmol), and pyridine (330 μL, 4.05 mmol) in DMSO (32 mL) as brown solid. Yield: 260 mg (1.51 mmol; 76%). C_10_H_8_N_2_O (M*_r_* 172.19). ^1^H NMR (DMSO-*d*_6_): δ = 8.44 (s, 1 H, C^2^*H*_Pyr_), 7.99 (dd, ^3^*J* = 6.7 Hz, ^5^*J* = 0.5 Hz, 1 H, C^6^*H*_Pyr_), 7.59–7.45 (m, 5 H, C*H*_Phen_), 6.52 (dd, ^3^*J* = 6.7 Hz, ^4^*J* = 1.0 Hz, 1 H, C^5^*H*_Pyr_) ppm. ^13^C NMR (DMSO-*d*_6_): δ = 159.8 (*C*^4^_Pyr_), 153.5 (*C*^6^_Pyr_), 152.0 (*C*^2^_Pyr_), 137.1 (*C*^1^_Phen_), 129.2 (*C*^3/5^_Phen_), 128.9 (*C*^4^_Phen_), 127.1 (*C*^2/6^_Phen_), 115.6 (*C*^5^_Pyr_) ppm. MS (ESI, 70 eV) *m*/*z* = 172.9 [M + H]^+^. Synthesis has been described by Abd El Kader et al. [26].

3-(2,5-Dimethoxyphenyl)pyrimidin-4(3*H*)-one (**2**): 3-(2,5-Dimethoxyphenyl)pyrimidin-4(3*H*)-one (**2**) was obtained from 4(3*H*)-pyrimidin-one (200 mg, 2.08 mmol), 2,5-dimethoxyphenylboronic acid (735 mg, 4.04 mmol), copper(I)-bromide (77.0 mg, 0.54 mmol), and pyridine (330 μL, 4.05 mmol) in DMSO (32 mL). Purification by column flash-chromatography on silica gel (gradient EE/PE) provided **2** as colorless solid. Yield: 202 mg (0.87 mmol, 42%). C_12_H_12_N_2_O_3_ (M*_r_* 232.24). ^1^H NMR (DMSO-*d*_6_): δ = 8.30 (dd, ^4^*J* = 0.9 Hz, ^5^*J* = 0.6 Hz, 1 H, C^2^*H*_Pyr_), 7.96 (dd, ^3^*J* = 6.7 Hz, ^5^*J* = 0.6 Hz, 1 H, C^6^*H*_Pyr_), 7.19–7.15 (m, 1 H, C^6^*H*_Phen_), 7.09–7.05 (m, 2 H, C^3^*H*_Phen,_ C^4^*H*_Phen_), 6.49 (dd, ^3^*J* = 6.7 Hz, ^5^*J* = 1.0 Hz, 1 H, C^5^*H*_Pyr_), 3.74 (s, 3 H, C^2^-OC*H*_3_), 3.71 (s, 3 H, C^5^-OC*H*_3_) ppm. ^13^C NMR (DMSO-*d*_6_): δ = 159.5 (*C*^4^_Pyr_), 153.5 (*C*^6^_Pyr_), 153.0 (*C*^2^_Phen_), 152.8 (*C*^2^_Pyr_), 148.2 (*C*^5^_Phen_), 126.0 (*C*^1^_Phen_), 115.70 (*C*^5^_Pyr_), 115.66 (*C*^3^_Phen_), 114.8 (*C*^4^_Phen_), 113.5 (*C*^6^_Phen_), 56.3 (C^2^_Phen_-O*C*H_3_), 55.7 (C^5^_Phen_-O*C*H_3_) ppm. MS (ESI, 70 eV) *m*/*z* = 172.9 [M+H]^+^.

3-(2,4-Dimethoxyphenyl)pyrimidin-4(3*H*)-one (3): 3-(2,4-Dimethoxyphenyl)pyrimidin-4(3*H*)-one (**3**) was obtained from 4(3*H*)-pyrimidin-one (206 mg, 2.14 mmol), 2,4-dimethoxyphenylboronic acid (732 mg, 4.02 mmol), copper(I)-bromide (65.0 mg, 0.45 mmol) and pyridine (330 μL, 4.05 mmol) in DMSO (32 mL). Purification by column flash-chromatography on silica gel (gradient EE/PE) provided **3** as light brown solid. Yield: 102 mg (0.44 mmol, 21%). C_12_H_12_N_2_O_3_ (M*_r_* 232.24). ^1^H NMR (DMSO-*d*_6_): δ = 8.25 (bs, 1 H, C^2^*H*_Pyr_), 7.95 (dd, ^3^*J* = 6.7 Hz, ^5^*J* = 0.4 Hz, 1 H, C^6^*H*_Pyr_), 7.27 (d, ^3^*J* = 8.6 Hz, 1 H, C^6^*H*_Phen_), 6.76 (d, ^4^*J* = 2.9 Hz, 1 H, C^3^*H*_Phen_), 6.64 (dd, ^3^*J* = 8.6 Hz, ^4^*J* = 2.6 Hz, 1 H, C^5^*H*_Phen_), 6.46 (dd, ^3^*J* = 6.7 Hz, ^5^*J* = 1.0 Hz, 1 H, C^5^*H*_Pyr_), 3.83 (s, 3 H, C^4^-OC*H*_3_), 3.76 (s, 3 H, C^2^-C*H*_3_) ppm. ^13^C NMR (DMSO-*d*_6_): δ = 161.7 (*C*^4^_Phen_), 160.3 (*C*^4^_Pyr_), 155.6 (*C*^2^_Phen_), 153.9 (*C*^6^_Pyr_), 153.8 (*C*^2^_Pyr_), 130.0 (*C*^6^_Phen_), 119.1 (*C*^1^_Phen_), 116.1 (*C*^5^_Pyr_), 105.6 (*C*^5^_Phen_), 99.8 (*C*^3^_Phen_), 55.5 (C^2^-O*C*H_3_), 55.1 (C^4^-O*C*H_3_) ppm. MS (ESI, HR): [M + H]^+^ *m*/*z* = calc.: 233.09207 , found: 233.09200.

3-(3,5-Dimethoxyphenyl)pyrimidin-4(3*H*)-one (4): 3-(3,5-Dimethoxyphenyl)pyrimidin-4(3*H*)-one (**4**) was obtained from 4(3*H*)-pyrimidin-one (193 mg, 2.01 mmol), 3,5-dimethoxyphenylboronic acid (727 mg, 3.99 mmol), copper(I)-bromide (60.0 mg, 0.42 mmol) and pyridine (330 μL, 4.05 mmol) in DMSO (32 mL). Purification by column flash-chromatography on silica gel (gradient EE/PE) provided **4** as colorless solid. Yield: 381 mg (1.64 mmol, 82%). C_12_H_12_N_2_O_3_ (M*_r_* 232.24). ^1^H NMR (DMSO-*d*_6_): δ = 8.41 (bs, 1 H, C^2^*H*_Pyr_), 7.98 (dd, ^3^*J* = 6.7 Hz, ^4^*J* = 0.5 Hz, 1 H, C^6^*H*_Pyr_), 6.67 (d, ^4^*J* = 2.2 Hz, 2 H, C^2/6^*H*_Phen_), 6.64 (t, ^4^*J* = 2.2 Hz, 1 H, C^4^*H*_Phen_), 6.50 (dd, ^3^*J* = 6.7 Hz, ^5^*J* = 1.0 Hz, 1 H, C^5^*H*_Pyr_), 3.78 (s, 6 H, OC*H*_3_) ppm. ^13^C NMR (DMSO-*d*_6_): δ = 161.1 (*C*^3/5^_Phen_), 160.1 (*C*^4^_Pyr_), 153.9 (*C*^6^_Pyr_), 152.4 (*C*^2^_Pyr_), 139.2 (*C*^1^_Phen_), 116.2 (*C*^5^_Pyr_), 106.1 (*C*^2/6^_Phen_), 101.3 (*C*^4^_Phen_), 56.1 (O*C*H_3_) ppm. MS (ESI, HR): [M + H]^+^ *m*/*z* = calc.: 232.09207, found: 233.09200.

### 4.6. General Procedure for the Synthesis of the Benzyl Pyrimidinone-Derivatives

4(3*H*)-Pyrimidinone and potassium hydroxide were dissolved in DMSO. Under nitrogen-atmosphere iron (nano) and the appropriate benzyl chloride were added. The mixture was heated at 110 °C for 2 h, decanted in ice and acidified with 1 M HCl. The aqueous phase was extracted with DCM. The organic phase was washed with water, dried over sodium sulfate and the solvent was removed under reduced pressure. The crude products were purified by column flash-chromatography on silica gel (gradient EE/PE).

3-(4-Methoxybenzyl)pyrimidin-4(3*H*)-one (**5**): 3-(4-Methoxybenzyl)pyrimidin-4(3*H*)-one (**5**) was obtained from 4(3*H*)-pyrimidinone (240 mg, 2.50 mmol), potassium hydroxide (213 mg, 3.79 mmol), nano-iron (7.2 mg, 0.13 mmol) and 1-(chloromethyl)-4-methoxybenzene (350 µL, 2.50 mmol) in DMSO (3 mL). Purification by column flash-chromatography on silica gel (gradient EE/PE) provided **5** as yellow solid. Yield: 270 mg (1.25 mmol, 50%). C_12_H_12_N_2_O_2_ (M*_r_* 216.24). ^1^H NMR (DMSO-*d*_6_): δ = 8.66 (s, 1 H, C^2^*H*_Pyr_), 7.90 (d, ^3^*J* = 6.6 Hz, 1 H, C^6^*H*_Pyr_), 7.31 (d, ^3^*J* = 6.6 Hz, 2 H, C^2/6^*H*_Benz_), 6.90 (d, ^3^*J* = 6.9 Hz, 2 H, C^3/5^*H*_Benz_), 6.40 (d, ^3^*J* = 6.4 Hz, 1 H, C^5^*H*_Pyr_), 5.01 (s, 2 H, C*H*_2_), 3.72 (s, 3 H, OC*H*_3_) ppm. ^13^C NMR (DMSO-*d*_6_): δ = 160.0 (*C*^4^_Pyr_), 159.9 (C^4^_Benz_), 153.6 (*C*^6^_Pyr_), 152.7 (*C*^2^_Pyr_), 129.6 (*C*^2/6^_Benz_), 128.4 (*C*^1^_Benz_), 115.2 (*C*^5^_Pyr_), 114.0 (*C*^3/5^_Benz_), 55.1 (O*C*H_3_), 48.4 (*C*H_2_) ppm. MS (ESI, 70 eV) *m*/*z* = 217.1 [M + H]^+^.

3-(4-Trifluoromethylbenzyl)pyrimidin-4(3*H*)-one (**6**): 3-(4-Trifluoromethylbenzyl)pyrimidin-4(3*H*)-one (**6**) was obtained from 4(3*H*)-pyrimidinone (247 mg, 2.57 mmol), potassium hydroxide (223 mg, 3.97 mmol), nano-iron (7.4 mg, 0.13 mmol) and 1-(chloromethyl)-4-(trifluoromethyl)benzene (370 µL, 2.50 mmol) in DMSO (2.5 mL). Purification by column flash-chromatography on silica gel (gradient EE/PE) provided **6** as yellow solid. Yield: 383 mg (1.51 mmol, 60%). C_12_H_9_F_3_N_2_O (M*_r_* 254.21). ^1^H NMR (DMSO-*d*_6_): δ = 8.71 (s, 1 H, C^2^*H*_Pyr_), 7.96 (dd, ^3^*J* = 6.6 Hz, ^4^*J* = 0.4 Hz, 1 H, C^6^*H*_Pyr_), 7.73 (d, ^3^*J* = 8.1 Hz, 2 H, C^3/5^*H*_Benz_), 7.52 (d, ^3^*J* = 8.1 Hz, 2 H, C^2/6^*H*_Benz_), 6.45 (dd, ^3^*J* = 6.6 Hz, ^5^*J* = 0.9 Hz, 1 H, C^5^*H*_Pyr_), 5.19 (s, 2 H, C*H*_2_), ppm. ^13^C NMR (DMSO-*d*_6_): δ = 160.0 (C^4^_Pyr_), 153.9 (*C*^6^_Pyr_), 153.0 (*C*^2^_Pyr_), 141.1 (*C*^1^_Benz_), 128.3 (*C*^2/6^_Benz_), 128.3 (d, ^2^*J*_CF_ = 31.4 Hz, *C*^4^_Benz_), 125.6 (q, ^3^*J*_CF_ = 3.9 Hz, *C*^3/5^_Benz_), 124.2 (d, ^1^*J*_CF_ = 271.5 Hz, *C*F_3_), 115.3 (*C*^5^_Pyr_), 48.8 (*C*H_2_) ppm. MS (ESI, 70 eV) *m*/*z* = 255.0 [M + H]^+^.

3-(2-Fluorobenzyl)pyrimidin-4(3*H*)-one (**7**): 3-(2-Fluorobenzyl)pyrimidin-4(3*H*)-one (**7**) was obtained from 4(3*H*)-pyrimidinone (247 mg, 2.57 mmol), potassium hydroxide (228 mg, 4.06 mmol), nano-iron (8.1 mg, 0.15 mmol) and 1-(chloromethyl)-2-fluorobenzene (300 µL, 2.50 mmol) in DMSO (3 mL). Purification by column flash-chromatography on silica gel (gradient EE/PE) provided **7** as yellow solid. Yield: 266 mg (1.31 mmol, 51%). C_11_H_9_FN_2_O (M*_r_* 204.20). ^1^H NMR (DMSO-*d*_6_): δ = 8.61 (s, 1 H, C^2^*H*_Pyr_), 7.94 (d, ^3^*J* = 6.6 Hz, 1 H, C^6^*H*_Pyr_), 7.39–7.33 (m, 1 H, C^3^*H*_Benz_), 7.24–7.15 (m, 3 H, C^4^*H*_Benz,_ C^5^*H*_Benz,_ C^6^*H*_Benz_), 6.42 (dd, ^3^*J* = 6.6 Hz, ^5^*J* = 0.8 Hz, 1 H, C^5^*H*_Pyr_), 5.14 (s, 2 H, C*H*_2_), ppm. ^13^C NMR (DMSO-*d*_6_): δ = 160.2 (d, ^1^*J*_CF_ = 245.5 Hz, *C*^2^_Benz_), 159.9 (C^4^_Pyr_), 153.8 (*C*^6^_Pyr_), 153.0 (d, ^5^*J*_CF_ = 2.2 Hz, *C*^2^_Pyr_), 130.0 (d, ^3^*J*_CF_ = 11.9 Hz, *C*^6^_Benz_), 130.0 (*C*^5^_Benz_), 124.6 (d, ^3^*J*_CF_ = 3.5 Hz, *C*^4^_Benz_), 123.0 (d, ^2^*J*_CF_ = 14.1 Hz, *C*^1^_Benz_), 115.6 (d, ^2^*J*_CF_ = 20.9 Hz, *C*^3^_Benz_), 115.2 (*C*^5^_Pyr_), 44.0 (d, ^3^*J*_CF_ = 4.0 Hz, *C*H_2_) ppm. MS (ESI, 70 eV) *m*/*z* = 205.1 [M + H]^+^.

### 4.7. General Procedure for the Synthesis of the Phenyl/benzyl-2-thioxo-Pyrimidinone-Derivatives

The appropriate phenyl/benzyl- pyrimidinone derivative and NaHCO_3_ were dissolved in a mixture of organic solvent and deionized water under vigorous stirring. Phenyl thionochloroformate was added dropwise and the mixture was stirred at room temperature for 5–16 h. After extraction with ethyl acetate the organic phase was washed three times with brine, dried over sodium sulfate and the solvent was removed under reduced pressure. The residue was then dissolved in methanol. TEA was added and the mixture was stirred at room temperature (or at 80 °C) for 4–16 h. The solvent was removed and the crude products were purified by column flash-chromatography on silica gel (gradient EE/PE).

3-Phenyl-2-thioxo-2,3-dihydropyrimidin-4(1*H*)-one (**8**): 3-Phenyl-2-thioxo-2,3-dihydropyrimidin-4(1*H*)-one (**8**) was obtained from **1** (176 mg, 1.03 mmol), NaHCO_3_ (512 mg, 6.09 mmol) and phenyl thionochloroformate (300 µL, 2.15 mmol) in diethyl ether (3 mL) and water (3 mL) in the first step. The residue was dissolved in methanol (6 mL) at room temperature (RT) and TEA (430 µL, 3.10 mmol) was added. Purification by column flash-chromatography on silica gel (gradient EE/PE) provided **8** as yellow solid. Yield: 95.8 mg (0.47 mmol, 46%). C_10_H_8_N_2_OS (M*_r_* 204.25). MS (ESI, 70 eV) *m*/*z* = 172.9 [M + H]^+^. Synthesis has been described by Nekkaa et al. [29].

3-(2,5-Dimethoxyphenyl)-2-thioxo-2,3-dihydropyrimidin-4(1*H*)-one (**9**): 3-(2,5-Dimethoxyphenyl)-2-thioxo-2,3-dihydropyrimidin-4(1*H*)-one (**9**) was obtained from **2** (244 mg, 1.05 mmol), NaHCO_3_ (536 mg, 6.38 mmol) and phenyl thionochloroformate (370 µL, 2.66 mmol) in diethyl ether (3 mL), ethyl acetate (3 mL) and water (3 mL) in the first step. The residue was dissolved in methanol (6 mL) at RT and TEA (451 µL, 3.25 mmol) was added. Purification by column flash-chromatography on silica gel (gradient EE/PE) provided **9** as light yellow solid. Yield: 209 mg (0.79 mmol, 79%). C_12_H_12_N_2_O_3_S (M*_r_* 264.30). ^1^H NMR (DMSO-*d*_6_): δ = 12.59 (d, ^3^*J* = 4.9 Hz, 1 H, N-*H*), 7.51 (dd, ^3^*J* = 7.6 Hz, ^3^*J* = 5.8 Hz, 1 H, C^6^*H*_Pyr_), 7.06 (d, ^3^*J* = 9.0 Hz, 1 H, C^3^*H*_Phen_), 6.95 (dd, ^3^*J* = 9.0 Hz, ^4^*J* = 3.0 Hz, 1 H, C^4^*H*_Phen_), 6.77 (d, ^4^*J* = 3.0 Hz, 1 H, C^6^*H*_Phen_), 5.99 (d, ^3^*J* = 7.6 Hz, 1 H, C^5^*H*_Pyr_), 3.71 (s, 3 H, C^5^-OC*H*_3_), 3.67 (s, 3 H, C^2^-OC*H*_3_) ppm. ^13^C NMR (DMSO-*d*_6_): δ = 178.0 (*C*^2^_Pyr_), 160.6 (*C*^4^_Pyr_), 153.7 (*C*^5^_Phen_), 148.8 (*C*^2^_Phen_), 141.8 (*C*^6^H_Pyr_), 128.5 (*C*^1^_Phen_), 116.1 (*C*^6^_Phen_), 114.8 (*C*^4^_Phen_), 113.7 (*C*^3^_Phen_), 105.0 (*C*^5^_Pyr_), 56.6 (C^2^-O*C*H_3_), 56.0 (C^5^-O*C*H_3_) ppm. MS (ESI, 70 eV) *m*/*z* = 265.0 [M + H]^+^, 529.0 [MMH]^+^.

3-(2,4-Dimethoxyphenyl)-2-thioxo-2,3-dihydropyrimidin-4(1*H*)-one (**10**): 3-(2,4-Dimethoxyphenyl)-2-thioxo-2,3-dihydropyrimidin-4(1*H*)-one (***10***) was obtained from **3** (125 mg, 0.54 mmol), NaHCO_3_ (278 mg, 3.31 mmol) and phenyl thionochloroformate (194 µL, 1.39 mmol) in diethyl ether (3 mL), ethyl acetate (3 mL) and water (3 mL) in the first step. The residue was dissolved in methanol (6 mL) at RT and TEA (237 µL, 1.71 mmol) was added. Purification by column flash-chromatography on silica gel (gradient EE/PE) provided ***10*** as light yellow solid. Yield: 114 mg (0.43 mmol, 78%). C_12_H_12_N_2_O_3_S (M*_r_* 264.30). ^1^H NMR (DMSO-*d*_6_): δ = 12.54 (s, 1 H, N-*H*), 7.48 (d, ^3^*J* = 7.6 Hz, 1 H, C^6^*H*_Pyr_), 7.00 (d, ^3^*J* = 8.6 Hz, 1 H, C^6^*H*_Phen_), 6.66 (d, ^4^*J* = 2.6 Hz, 1 H, C^3^*H*_Phen_), 6.56 (dd, ^3^*J* = 8.6 Hz, ^4^*J* = 2.6 Hz, 1 H, C^5^*H*_Phen_), 5.97 (d, ^3^*J* = 7.6 Hz, 1 H, C^5^*H*_Pyr_), 3.80 (s, 3 H, C^2^-OC*H*_3_), 3.70 (s, 3 H, C^4^-OC*H*_3_) ppm. ^13^C NMR (DMSO-*d*_6_): δ = 178.1 (*C*^2^_Pyr_), 160.4 (*C*^4^_Pyr_), 160.4 (*C*^2^_Phen_), 154.9 (*C*^4^_Phen_), 141.2 (*C*^6^_Pyr_), 129.9 (*C*^6^_Phen_), 120.5 (*C*^1^_Phen_), 105.1 (*C*^5^_Phen_), 104.4 (*C*^5^_Pyr_), 99.3 (*C*^3^_Phen_), 55.7 (C^4^-O*C*H_3_), 55.4 (C^2^-O*C*H_3_) ppm. MS (ESI, 70 eV) *m*/*z* = 265.0 [M + H]^+^, 529.0 [MMH]^+^.

3-(3,5-Dimethoxyphenyl)-2-thioxo-2,3-dihydropyrimidin-4(1*H*)-one (**11**): 3-(3,5-Dimethoxyphenyl)-2-thioxo-2,3-dihydropyrimidin-4(1*H*)-one (***11***) was obtained from **4** (350 mg, 1.51 mmol), NaHCO_3_ (759 mg, 9.03 mmol) and phenyl thionochloroformate (529 µL, 3.80 mmol) in diethyl ether (6 mL) and water (6 mL) in the first step. The residue was dissolved in methanol (15 mL) at RT and TEA (645 µL, 4.65 mmol) was added. Purification by column flash-chromatography on silica gel (gradient EE/PE) provided ***11*** as yellow solid. Yield: 280 mg (1.06 mmol, 70%). C_12_H_12_N_2_O_3_S (M*_r_* 264.30). ^1^H NMR (DMSO-*d*_6_): δ = 12.62 (d, ^3^*J* = 3.1 Hz, 1 H, N-*H*), 7.50 (d, ^3^*J* = 7.6 Hz, ^3^*J* = 5.1 Hz, 1 H, C^6^*H*_Pyr_), 6.52 (t, ^4^*J* = 2.3 Hz, 1 H, C^4^*H*_Phen_), 6.38 (d, ^4^*J* = 2.3 Hz, 2 H, C^2/6^*H*_Phen_), 6.01 (d, ^3^*J* = 7.6 Hz, 1 H, C^5^*H*_Pyr_), 3.73 (s, 6 H, C^3/5^-OC*H*_3_) ppm. ^13^C NMR (DMSO-*d*_6_): δ = 177.5 (*C*^2^_Pyr_), 160.8 (*C*^3/5^_Phen_), 160.5 (*C*^4^_Pyr_), 141.2 (*C*^6^_Pyr_), 140.7 (*C*^1^_Phen_), 106.9 (*C*^2/6^_Phen_), 104.9 (*C*^5^_Pyr_), 100.0 (*C*^4^_Phen_), 55.4 (C^3/5^-O*C*H_3_) ppm. MS (ESI, 70 eV) *m*/*z* = 265.0 [M + H]^+^, 529.0 [MMH]^+^.

3-(4-Methoxybenzyl)-2-thioxo-2,3-dihydropyrimidin-4(1*H*)-one (**12**): 3-(4-Methoxybenzyl)-2-thioxo-2,3-dihydropyrimidin-4(1*H*)-one (**12**) was obtained from **5** (251 mg, 1.16 mmol), NaHCO_3_ (586 mg, 6.97 mmol) and phenyl thionochloroformate (380 µL, 2.73 mmol) in EE (5 mL) and water (5 mL) in the first step. The residue was dissolved in methanol (10 mL) and TEA (429 µL, 3.09 mmol) was added. The mixture was stirred at 80 °C for 9 h. Purification by column flash-chromatography on silica gel (gradient EE/PE) provided **12** as colorless solid. Yield: 65.5 mg (0.26 mmol, 23%). C_12_H_12_N_2_O_2_S (M*_r_* 248.30). ^1^H NMR (DMSO-*d*_6_): δ = 12.63 (bs, 1 H, N-*H*), 7.47 (dd, ^3^*J* = 7.5 Hz, ^3^*J* = 5.7 Hz, 1 H, C^6^*H*_Pyr_), 7.28 (d, ^3^*J* = 8.7 Hz, 2 H, C^2/6^*H*_Benz_), 6.85 (d, ^3^*J* = 8.8 Hz, 2 H, C^3/5^*H*_Benz_), 5.97 (dd, ^3^*J* = 7.5 Hz, ^4^*J* = 0.7 Hz, 1 H, C^5^*H*_Pyr_), 5.43 (s, 2 H, C*H*_2_), 3.71 (s, 3 H, OC*H*_3_) ppm. ^13^C NMR (DMSO-*d*_6_): δ = 176.9 (*C*^2^_Pyr_), 160.4 (*C*^4^_Pyr_), 158.1 (*C*^4^_Benz_), 140.9 (*C*^6^_Pyr_), 129.1 (*C*^2/6^_Benz_), 128.1 (*C*^1^_Benz_), 113.6 (*C*^3/5^_Benz_), 104.4 (*C*^5^_Pyr_), 55.1 (O*C*H_3_), 47.7 (*C*H_2_) ppm. MS (ESI, 70 eV) *m*/*z* = 248.9 [M + H]^+^.

3-(4-Trifluoromethylbenzyl)-2-thioxo-2,3-dihydropyrimidin-4(1*H*)-one (**13**): 3-(4-Trifluoromethylbenzyl)-2-thioxo-2,3-dihydropyrimidin-4(1*H*)-one (**13**) was obtained from **6** (298 mg, 1.17 mmol), NaHCO_3_ (597 mg, 7.11 mmol) and phenyl thionochloroformate (380 µL, 2.73 mmol) in EE (5 mL) and water (5 mL) in the first step. The residue was dissolved in methanol (10 mL) and TEA (433 µL, 3.12 mmol) was added. The mixture was stirred at 80 °C for 4 h. Purification by column flash-chromatography on silica gel (gradient EE/PE) provided **13** as yellow solid. Yield: 142 mg (0.50 mmol, 42%). C_12_H_9_F_3_N_2_OS (M*_r_* 286.27). ^1^H NMR (DMSO-*d*_6_): δ = 12.75 (d, ^3^*J* = 5.2 Hz, 1 H, N-*H*), 7.67 (d, ^3^*J* = 8.1 Hz, 2 H, C^3/5^*H*_Benz_), 7.54 (dd, ^3^*J* = 7.5 Hz, ^3^*J* = 5.8 Hz, 1 H, C^6^*H*_Pyr_), 7.47 (d, ^3^*J* = 8.0 Hz, 2 H, C^2/6^*H*_Benz_), 6.02 (dd, ^3^*J* = 7.5 Hz, ^4^*J* = 1.4 Hz, 1 H, C^5^*H*_Pyr_), 5.58 (s, 2 H, C*H*_2_) ppm. ^13^C NMR (DMSO-*d*_6_): δ = 177.1 (*C*^2^_Pyr_), 160.4 (*C*^4^_Pyr_), 141.3 (*C*^6^_Pyr_), 141.1 (*C*^1^_Benz_), 127.9 (d, ^2^*J*_CF_ = 35.2 Hz, *C*^4^_Benz_), 128.1 (*C*^1^_Benz_), 127.7 (*C*^2/6^_Benz_), 125.2 (q, ^3^*J*_CF_ = 3.8 Hz, *C*^3/5^_Benz_), 124.3 (d, ^1^*J*_CF_ = 272.3 Hz, *C*F_3_), 104.3 (*C*^5^_Pyr_), 48.2 (*C*H_2_) ppm. MS (ESI, 70 eV) *m*/*z* = 286.9 [M + H]^+^.

3-(2-Fluorobenzyl)-2-thioxo-2,3-dihydropyrimidin-4(1*H*)-one (**14**): 3-(2-Fluorobenzyl)-2-thioxo-2,3-dihydropyrimidin-4(1*H*)-one (**14**) was obtained from **7** (223 mg, 1.09 mmol), NaHCO_3_ (552 mg, 6.57 mmol) and phenyl thionochloroformate (360 µL, 2.59 mmol) in EE (5 mL) and water (5 mL) in the first step. The residue was dissolved in methanol (8 mL) and TEA (402 µL, 2.90 mmol) was added. The mixture was stirred at 80 °C for 16 h. Purification by column flash-chromatography on silica gel (gradient EE/PE) provided **14** as colorless solid. Yield: 55.4 mg (0.23 mmol, 22%). C_11_H_9_FN_2_OS (M*_r_* 236.26). ^1^H NMR (DMSO-*d*_6_): δ = 12.74 (d, ^3^*J* = 5.4 Hz, 1 H, N-*H*), 7.54 (dd, ^3^*J* = 7.5 Hz, ^3^*J* = 5.8 Hz, 1 H, C^6^*H*_Pyr_), 7.32–7.26 (m, 1 H, C^4^*H*_Benz_), 7.22–7.12 (m, 1 H, C^3^*H*_Benz_), 7.11 (td, ^3^*J* = 7.5 Hz, ^4^*J* = 1.0 Hz, 1 H, C^5^*H*_Benz_), 7.00–6.97 (m, 1 H, C^6^*H*_Benz_), 6.02 (dd, ^3^*J* = 7.5 Hz, ^4^*J* = 1.3 Hz, 1 H, C^5^*H*_Pyr_), 5.53 (s, 2 H, C*H*_2_) ppm. ^13^C NMR (DMSO-*d*_6_): δ = 177.1 (*C*^2^_Pyr_), 160.3 (*C*^4^_Pyr_), 159.9 (d, ^1^*J*_CF_ = 244.3 Hz, *C*^2^_Benz_), 141.3 (*C*^6^_Pyr_), 128.8 (d, ^3^*J*_CF_ = 8.1 Hz, *C*^4^_Benz_), 127.0 (d, ^3^*J*_CF_ = 4.1 Hz, *C*^6^_Benz_), 124.4 (d, ^4^*J*_CF_ = 3.4 Hz, *C*^5^_Benz_), 122.9 (d, ^2^*J*_CF_ = 14.1 Hz, *C*^1^_Benz_), 115.2 (d, ^2^*J*_CF_ = 21.0 Hz, *C*^3^_Benz_), 104.2 (*C*^5^_Pyr_), 42.8 (d, ^3^*J*_CF_ = 5.5 Hz, *C*H_2_) ppm. MS (ESI, 70 eV) *m*/*z* = 237.0 [M + H]^+^.

### 4.8. Synthesis of the Benzothiazole–Linker

2-Chloro-*N*-(6-(trifluoromethyl)benzo[d]thiazol-2-yl)acetamide (**15**): A solution of 2-amino-6-(trifluoromethyl)benzo[*d*]thiazole (542 mg, 2.48 mmol) and TEA (412 µL, 2.73 mmol) in DCM (4.5 mL) was added dropwise to a solution of 2-chloracetyl chloride (217 µL, 2.73 mmol) in DCM (1.5 mL). The mixture was then stirred for 24 h at rt. The solvent was concentrated under reduced pressure. The product was filtered, washed with water and recrystallized from EtOH/H_2_O to give light brownish crystals. Yield: 585 mg (1.99 mmol, 80%). C_10_H_6_ClF_3_N_2_OS (M*_r_* 294,68). ^1^H NMR (DMSO-*d*_6_): δ = 12.96 (s, 1 H, N*H*), 8.52 (d, ^4^*J* = 1.6 Hz, 1 H, C^7^*H*_Bnth_), 7.94 (d, ^3^*J* = 8.5 Hz, 1 H, C^4^*H*_Bnth_), 7.75 (dd, ^3^*J* = 8.5 Hz, ^4^*J* = 1.6 Hz, 1 H, C^5^*H*_Bnth_), 4.50 (s, 2 H, C*H*_2_-Cl) ppm. ^13^C NMR (DMSO-*d*_6_): δ = 166.4 (*C*=O), 160.8 (C^2^_Bnth_), 151.2 (*C*^3a^_Bnth_), 132.0 (*C*^7a^_Bnth_)_,_ 124.5 (d, ^1^*J*_CF_ = 271.8 Hz, *C*F_3_), 123.9 (d, ^2^*J*_CF_ = 31.9 Hz, C^6^_Bnth_), 123.0 (d, ^3^*J*_CF_ = 3.6 Hz, *C*^5^H_Bnth_), 121.2 (*C*^4^H_Bnth_), 120.0 (d, ^3^*J*_CF_ = 4.0 Hz, *C*^7^H_Bnth_), 42.5 (*C*H_2_-Cl) ppm. MS (ESI, 70 eV) *m*/*z* = 294.8 [M + H]^+^. Synthesis has been described by García-Reyes et al. [15].

### 4.9. General Procedure for the Synthesis of the IWP Derived Derivatives

Under nitrogen atmosphere the appropriate phenyl/benzyl-2-thioxo-pyrimidinone-derivative and **15** were dissolved in DMF. TEA was added and the mixture was heated to 80 °C for 2 h. After quenching with water, the reaction mixture was extracted with ethyl acetate. The organic phase was washed respectively with deionized water and brine three times and dried over sodium sulfate. The solvent was removed under reduced pressure and the crude product was purified by column flash-chromatography on silica gel (gradient EE/PE) or by recrystallization from EtOH/H_2_O.

2-((3-Phenyl-4-oxo-3,4-dihydropyrimidin-2-yl)thio)-*N*-(6-(trifluoromethyl)benzo-[d]thiazol-2-yl)acetamide (**16**): 2-((3-Phenyl-4-oxo-3,4-dihydropyrimidin-2-yl)thio)-*N*-(6-(trifluoromethyl)benzo-[*d*]thiazol-2-yl)acetamide (***16***) was obtained from ***15*** (146 mg, 0.50), **8** (131 mg, 0.50 mmol), and TEA (0.2 mL, 1.44 mmol). Purification by chromatography and recrystallization from EtOH/H_2_O provided ***16*** as light yellow solid. Yield: 102 mg (0.22 mmol, 44%). C_20_H_13_F_3_N_4_O_2_S_2_ (M*_r_* 462.47). HPLC (purity): 100%. ^1^H NMR (DMSO-*d*_6_): δ = 12.87 (s, 1 H, N*H*), 8.49 (s, 1 H, C^7^*H*_Bnth_), 7.92 (d, ^3^*J* = 8.6 Hz, 1 H, C^4^*H*_Bnth_), 7.85 (d, ^3^*J* = 6.6 Hz, 1 H, C^6^*H*_Pyr_), 7.75 (d, ^3^*J* = 8.6 Hz, 1 H, C^5^*H*_Bnth_), 7.63–7.58 (m, 3 H, C^4^*H*_Phen_, C^3^*H*_Phen_), 7.45–7.43 (m, 2 H, C^2^*H*_Phen_), 6.29 (d, ^3^*J* = 6.6 Hz, 1 H, C^5^*H*_Pyr_), 4.21 (s, 2 H, C*H*_2_-C=O) ppm. ^13^C NMR (DMSO-*d*_6_): δ = 167.3 (CH_2_-*C*=O), 161.0 (*C*^2^_Bnth_), 160.7 (*C*^4^_Pyr_), 152.3 (*C*^6^_Pyr_), 151.3 (*C*^3a^_Bnth_), 135.5 (*C*^1^_Phen_), 132.0 (*C*^7a^_Bnth_), 130.1 (*C*^4^H_Phen_), 129.7 (*C*^3^H_Phen_), 128.7 (*C*^2^H_Phen_), 124.5 (d, ^1^*J*_CF_ = 271.7 Hz, CF_3_), 123.8 (d, ^2^*J*_CF_ = 31.9 Hz, C^6^_Bnth_), 123.0 (d, ^3^*J*_CF_ = 3.1 Hz, *C*^5^H_Bnth_), 121.0 (*C*^4^H_Bnth_), 119.9 (d, ^3^*J*_CF_ = 4.4 Hz, *C*^7^H_Bnth_) 111.0 (*C*^5^_Pyr_), 36.0 (*C*H_2_-C=O) ppm. MS (ESI, 70 eV) *m*/*z* = 463.0 [M + H]^+^. IR: $\tilde{v}$ = 3132, 3036, 2989, 1549, 1478, 1314, 1152, 1117, 1078, 833, 826, 698, 677, cm^−1^.

2-((3-(2,5-Dimethoxyphenyl)-4-oxo-3,4-dihydropyrimidin-2-yl)thio)-*N*-(6-(trifluoromethyl)benzo[d]thiazol-2-yl)acetamide (**17**):2-((3-(2,5-Dimethoxyphenyl)-4-oxo-3,4-dihydropyrimidin-2-yl)thio)-*N*-(6-(trifluoro-methyl)benzo[*d*]thiazol-2-yl)acetamide (***17***) was obtained from ***15*** (202 mg, 0.69 mmol), **9** (173 mg, 0.65 mmol), and TEA (0.27 mL, 1.95 mmol). Recrystallization from EtOH/H_2_O provided ***17*** as light yellow solid. Yield: 243 mg (0.46 mmol, 71%). C_22_H_17_F_3_N_4_O_4_S_2_ (M*_r_* 522.52). HPLC (purity): 98%. ^1^H NMR (DMSO-*d*_6_): δ = 12.87 (s, 1 H, N*H*), 8.50 (s, 1 H, C^7^*H*_Bnth_), 7.92 (d, ^3^*J* = 8.4 Hz, 1 H, C^4^*H*_Bnth_), 7.83 (d, ^3^*J* = 6.6 Hz, 1 H, C^6^*H*_Pyr_), 7.75 (d, ^3^*J* = 8.4 Hz, 1 H, C^5^*H*_Bnth_), 7.22 (d, ^3^*J* = 9.1 Hz, 1 H, C^3^*H*_Phen_), 7.13 (dd, ^3^*J* = 9.1 Hz, ^4^*J* = 2.9 Hz, 1 H, C^4^*H*_Phen_), 7.04 (d, ^4^*J* = 2.9 Hz, 1 H, C^6^*H*_Phen_), 6.25 (d, ^3^*J* = 6.6 Hz, 1 H, C^5^*H*_Pyr_), 4.20 (s, 2 H, C*H*_2_-C=O), 3.753 (s, 3H, C*H*_3_-OC^2^_Phen_), 3.745 (s, 3H, C*H*_3_-OC^5^_Phenyl_) ppm. ^13^C NMR (DMSO-*d*_6_): δ = 167.4 (CH_2_-*C*=O), 163.1 (*C*^2^_Pyr_), 161.0 (*C*^2^_Bnth_), 160.2 (*C*^4^_Pyr_), 153.4 (*C*^5^_Phen_), 152.4 (*C*^6^_Pyr_), 151.3 (*C*^3a^_Bnth_), 148.6 (*C*^2^_Phen_), 132.0 (*C*^7a^_Bnth_), 124.6 (d, ^1^*J*_CF_ = 271.9 Hz, CF_3_), 124.1 (*C*^1^_Phen_), 123.8 (d, ^2^*J*_CF_ = 31.8 Hz, C^6^_Bnth_), 123.0 (d, ^3^*J*_CF_ = 3.3 Hz, *C*^5^H_Bnth_), 121.1 (*C*^4^H_Bnth_), 120.0 (d, ^3^*J*_CF_ = 4.1 Hz, *C*^7^H_Bnth_), 116.8 (*C*^4^H_Phen_), 115.6 (*C*^6^H_Phen_), 113.9 (*C*^3^H_phen_), 110.8 (*C*^5^_Pyr_), 56.4 (*C*H_3_-OC^5^_Phen_), 55.8 (*C*H_3_-OC^2^_Phen_), 36.0 (*C*H_2_-C=O) ppm. MS (ESI, HR): [M + H]^+^ *m*/*z* = calc.: 523.072159, found.: 532.07159. IR: $\tilde{v}$ = 3138, 3059, 2968, 2930, 1665, 1553, 1510, 1481, 1317, 1269, 1153, 1101, 1078, 1038, 839, 831, 677 cm^−1^.

2-((3-(2,4-Dimethoxyphenyl)-4-oxo-3,4-dihydropyrimidin-2-yl)thio)-*N*-(6-(trifluoromethyl)benzo[d]thiazol-2-yl)acetamide (**18**): 2-((3-(2,4-Dimethoxyphenyl)-4-oxo-3,4-dihydropyrimidin-2-yl)thio)-*N*-(6-(trifluoro-methyl)benzo[*d*]thiazol-2-yl)acetamide (***18***) was obtained from ***15*** (117 mg, 0.40 mmol), **10** (93.3 mg, 0.35 mmol), and TEA (158 µL, 1.14 mmol). Purification by chromatography provided ***18*** as light yellow solid. Yield: 109 mg (0.21 mmol, 59%). C_22_H_17_F_3_N_4_O_4_S_2_ (M*_r_* 522.52). HPLC (purity): 97%. ^1^H NMR (DMSO-*d*_6_): δ = 12.87 (s, 1 H, N*H*), 8.50 (bs, 1 H, C^7^*H*_Bnth_), 7.92 (d, ^3^*J* = 8.5 Hz, 1 H, C^4^*H*_Bnth_), 7.82 (d, ^3^*J* = 6.6 Hz, 1 H, C^6^*H*_Pyr_), 7.75 (dd, ^3^*J* = 8.5 Hz, ^4^*J* = 1.5 Hz, 1 H, C^5^*H*_Bnth_), 7.26 (d, ^3^*J* = 8.6 Hz, 1 H, C^6^*H*_Phen_), 6.80 (d, ^4^*J* = 2.5 Hz, 1 H, C^3^*H*_Phen_), 6.68 (dd, ^3^*J* = 8.6 Hz, ^4^*J* = 2.5 Hz, 1 H, C^5^*H*_Phen_), 6.23 (d, ^3^*J* = 6.6 Hz, 1 H, C^5^*H*_Pyr_), 4.18 (d, ^4^*J* = 1.7 Hz, 2 H, C*H*_2_-C=O), 3.85 (s, 3H, C*H*_3_-OC^4^_Phen_), 3.78 (s, 3H, C*H*_3_-OC^2^_Phen_) ppm. ^13^C NMR (DMSO-*d*_6_): δ = 167.5 (CH_2_-*C*=O), 163.9 (*C*^2^_Pyr_), 162.1 (*C*^4^_Phen_), 161.0 (*C*^2^_Bnth_), 160.5 (*C*^4^_Pyr_), 155.6 (*C*^2^_Phen_), 152.4 (*C*^6^H_Pyr_), 151.3 (*C*^3a^_Bnth_), 132.0 (*C*^7a^_Bnth_), 130.6 (*C*^6^H_Phen_), 124.6 (d, ^1^*J*_CF_ = 271.8 Hz, CF_3_), 123.8 (d, ^2^*J*_CF_ = 31.9 Hz, C^6^_Bnth_), 123.0 (d, ^3^*J*_CF_ = 3.6 Hz, *C*^5^H_Bnth_), 121.1 (*C*^4^H_Bnth_), 120.0 (d, ^3^*J*_CF_ = 4.2 Hz, *C*^7^H_Bnth_), 116.5 (*C*^1^_Phen_), 110.87(*C*^5^_Pyr_), 105.8 (*C*^5^H_Phen_), 99.6 (*C*^3^_Phen_), 56.1 (*C*H_3_-OC^2^_Phen_), 55.7 (*C*H_3_-OC^4^_Phen_), 35.9 (*C*H_2_-C=O) ppm. MS (ESI, 70 eV) [M+H]^+^ m/z = calc.: 523.072159, found.: 532.07156. IR: $\tilde{v}$ = 3067, 2976, 2941, 1688, 1541, 1485, 1317, 1269, 1159, 1115, 1082, 1028, 824, 718, 677cm^−1^.

2-((3-(3,5-Dimethoxyphenyl)-4-oxo-3,4-dihydropyrimidin-2-yl)thio)-*N*-(6-(trifluoromethyl)benzo[d]thiazol-2-yl)acetamide (**19**): 2-((3-(3,5-Dimethoxyphenyl)-4-oxo-3,4-dihydropyrimidin-2-yl)thio)-*N*-(6-(trifluoro-methyl)benzo[*d*]thiazol-2-yl)acetamide (***19***) was obtained from **15** (223 mg, 0.76 mmol), ***11*** (185 mg, 0.70 mmol), and TEA (291 µL, 2.10 mmol). Purification by chromatography provided *19* as light yellow solid. Yield: 248 mg (0.48 mmol, 68%). C_22_H_17_F_3_N_4_O_4_S_2_ (M*_r_* 522.52). HPLC (purity): 98%. ^1^H NMR (DMSO-*d*_6_): δ = 12.88 (s, 1 H, N*H*), 8.50 (bs, 1 H, C^7^*H*_Bnth_), 7.92 (d, ^3^*J* = 8.5 Hz, 1 H, C^4^*H*_Bnth_), 7.83 (d, ^3^*J* = 6.6 Hz, 1 H, C^6^*H*_Pyr_), 7.75 (dd, ^3^*J* = 8.5 Hz, ^4^*J* = 1.1 Hz, 1 H, C^5^*H*_Bnth_), 6.70 (t, ^4^*J* = 2.1 Hz, 1 H, C^4^*H*_Phen_), 6.64 (d, ^4^*J* = 2.1 Hz, 2 H, C^2/6^*H*_Phen_), 6.26 (d, ^3^*J* = 6.6 Hz, 1 H, C^5^*H*_Pyr_), 4.20 (s, 2 H, C*H*_2_-C=O), 3.79 (s, 6H, C*H*_3_-OC) ppm. ^13^C NMR (DMSO-*d*_6_): δ = 167.5 (CH_2_-*C*=O), 162.5 (*C*^2^_Pyr_), 161.1 (*C*^3/5^_Phen_), 161.0 (*C*^2^_Bnth_), 160.5 (*C*^4^_Pyr_), 152.2 (*C*^6^H_Pyr_), 151.3 (*C*^3a^_Bnth_), 137.2 (*C*^1^_Phen_), 132.0 (*C*^7a^_Bnth_), 124.6 (d, ^1^*J*_CF_ = 272.3 Hz, CF_3_), 123.8 (d, ^2^*J*_CF_ = 31.7 Hz, C^6^_Bnth_), 123.0 (d, ^3^*J*_CF_ = 3.5 Hz, *C*^5^H_Bnth_), 121.1 (*C*^4^H_Bnth_), 120.0 (d, ^3^*J*_CF_ = 4.2 Hz, *C*^7^H_Bnth_), 111.0 (*C*^5^_Pyr_), 106.9 (*C*^2/6^H_Phen_), 101.7 (*C*^4^H_Phen_), 55.7 (*C*H_3_-OC^3/5^_Phen_), 36.0 (*C*H_2_-C=O) ppm. MS (ESI, 70 eV) *m*/*z* = 522.9 [M+H]^+^. IR: $\tilde{v}$ = 3070, 2957, 1668, 1511, 1498, 1317, 1269, 1153, 1111, , 839, 826, 679 cm^−1^.

2-((3-(4-Methoxybenzyl)-4-oxo-3,4-dihydropyrimidin-2-yl)thio)-*N*-(6-(trifluoromethyl)benzo[d]thiazol-2-yl)acetamide (**20**):2-((3-(4-Methoxybenzyl)-4-oxo-3,4-dihydropyrimidin-2-yl)thio)-*N*-(6-(trifluoromethyl)benzo[*d*]thiazol-2-yl)acetamide (***20***) was obtained from ***15*** (72.2 mg, 0.25 mmol), ***12*** (57.8 mg, 0.23 mmol), and TEA (96.9 µL, 0.70 mmol); ***20*** was washed with DCM and collected as colorless solid. Yield: 46.9 mg (92.6 µmol, 40%). C_22_H_17_F_3_N_4_O_2_S_2_ (M*_r_* 506.52). HPLC (purity): 100%. ^1^H NMR (DMSO-*d*_6_): δ = 12.95 (s, 1 H, N*H*), 8.49 (bs, 1 H, C^7^*H*_Bnth_), 7.92 (d, ^3^*J* = 8.4 Hz, 1 H, C^4^*H*_Bnth_), 7.78 (d, ^3^*J* = 6.5 Hz, 1 H, C^6^*H*_Pyr_), 7.74 (d, ^3^*J* = 8.6 Hz, 1 H, C^5^*H*_Bnth_), 7.25 (d, ^3^*J* = 8.5 Hz, 2 H, C^2/6^*H*_Benz_), 6.92 (d, ^3^*J* = 8.5 Hz, 2 H, C^3/5^*H*_Benz_), 6.25 (d, ^3^*J* = 6.4 Hz, 1 H, C^5^*H*_Pyr_), 5.21 (s, 2 H, N-C*H*_2_), 4.32 (s, 2 H, C*H*_2_-C=O) ppm. ^13^C NMR (DMSO-*d*_6_): δ = 167.4 (CH_2_-*C*=O), 162.1 (*C*^2^_Pyr_), 161.2 (*C*^2^_Bnth_), 160.9 (*C*^4^_Pyr_), 158.8 (*C*^4^_Benz_), 152.1 (*C*^6^H_Pyr_), 151.4 (*C*^3a^_Bnth_), 132.0 (*C*^7a^_Bnth_), 128.8 (*C*^2/6^_Benz_), 126.8 (*C*^1^_Benz_), 124.6 (d, ^1^*J*_CF_ = 271.8 Hz, CF_3_), 123.8 (d, ^2^*J*_CF_ = 32.3 Hz, C^6^_Bnth_), 123.0 (d, ^3^*J*_CF_ = 3.3 Hz, *C*^5^_Bnth_), 121.0 (*C*^4^_Bnth_), 120.0 (d, ^3^*J*_CF_ = 4.2 Hz, *C*^7^_Bnth_), 114.0 (*C*^3/5^_Benz_), 110.2 (*C*^5^_Pyr_), 55.1 (O*C*H_3_), 46.5 (N-*C*H_2_), 36.0 (*C*H_2_-C=O) ppm. MS (ESI, 70 eV) *m*/*z* = 288.9 [C_14_H_13_N_2_O_3_S]^+^. IR: $\tilde{v}$ = 2936, 2849, 1649, 1560, 1497, 1373, 1319, 1256, 1244, 1159, 1111, 1084, 986, 827, 799, 677 cm^−1^.

2-((3-(4-Trifluoromethylbenzyl)-4-oxo-3,4-dihydropyrimidin-2-yl)thio)-*N*-(6-(trifluoromethyl)benzo[d]thiazol-2-yl)acetamide (**21**): 2-((3-(4-Trifluoromethylbenzyl)-4-oxo-3,4-dihydropyrimidin-2-yl)thio)-*N*-(6-(trifluoromethyl)benzo[*d*]thiazol-2-yl)acetamide (***21***) was obtained from ***15*** (105 mg, 0.36 mmol), ***13*** (97.5 mg, 0.34 mmol) and TEA (142 µL, 1.02 mmol). Purification by chromatography provided ***21*** as colorless solid. Yield: 71.1 mg (0.13 mmol, 38%). C_22_H_14_F_6_N_4_O_2_S_2_ (M*_r_* 544.49). HPLC (purity): 100%. ^1^H NMR (DMSO-*d*_6_): δ = 12.94 (s, 1 H, N*H*), 8.50 (bs, 1 H, C^7^*H*_Bnth_), 7.92 (d, ^3^*J* = 8.5 Hz, 1 H, C^4^*H*_Bnth_), 7.83 (d, ^3^*J* = 6.5 Hz, 1 H, C^6^*H*_Pyr_), 7.76–7.74 (m, 3 H, C^5^*H*_Bnth_, C^3/5^*H*_Benz_), 7.49 (d, ^3^*J* = 8.1 Hz, 2 H, C^2/6^*H*_Benz_), 6.29 (d, ^3^*J* = 6.5 Hz, 1 H, C^5^*H*_Pyr_), 5.38 (s, 2 H, N-C*H*_2_), 4.33 (s, 2 H, C*H*_2_-C=O) ppm. ^13^C NMR (DMSO-*d*_6_): δ = 167.2 (CH_2_-*C*=O), 162.1 (*C*^2^_Pyr_), 161.0 (*C*^2^_Bnth_), 160.9 (*C*^4^_Pyr_), 152.3 (*C*^6^H_Pyr_), 151.3 (*C*^3a^_Bnth_), 139.8 (*C*^1^_Benz_), 132.0 (*C*^7a^_Bnth_), 128.2 (d, ^2^*J*_CF_ = 31.8 Hz, *C*^4^_Benz_), 127.6 (*C*^2/6^_Benz_), 125.7 (d, ^3^*J*_CF_ = 3.7 Hz, *C*^3/5^_Benz_), 124.6 (d, ^1^*J*_CF_ = 272.5 Hz, CF_3_), 124.2 (d, ^1^*J*_CF_ = 271.4 Hz, *C*F_3_-C^4^_Benz_), 123.9 (d, ^2^*J*_CF_ = 32.1 Hz, C^6^_Bnth_), 123.1 (d, ^3^*J*_CF_ = 3.1 Hz, *C*^5^_Bnth_), 121.1 (*C*^4^_Bnth_), 120.0 (d, ^3^*J*_CF_ = 4.2 Hz, *C*^7^_Bnth_), 110.3 (*C*^5^_Pyr_), 46.7 (N-*C*H_2_), 35.9 (*C*H_2_-C=O) ppm. MS (ESI, 70 eV) *m*/*z* = 326.9 [C_14_H_10_F_3_N_2_O_2_S]^+^. IR: $\tilde{v}$ = 3065, 2978, 2922, 1655, 1562, 1493, 1319, 1153, 1119, 1111, 1066, 852, 799, 677, cm^−1^.

2-((3-(2-Fluoromethylbenzyl)-4-oxo-3,4-dihydropyrimidin-2-yl)thio)-N-(6-(trifluoro-methyl)benzo[d]thiazol-2-yl)acetamide (**22**): 2-((3-(2-Fluoromethylbenzyl)-4-oxo-3,4-dihydropyrimidin-2-yl)thio)-*N*-(6-(trifluoro-methyl)benzo[*d*]thiazol-2-yl)acetamide (***22***) was obtained from ***15*** (67.7 mg, 0.23 mmol), ***14*** (48.5 mg, 0.21 mmol) and TEA (85.3 µL, 0.62 mmol). Purification by chromatography provided ***22*** as colorless solid. Yield: 82.2 mg (0.17 mmol, 81%). C_21_H_14_F_4_N_4_O_2_S_2_ (M*_r_* 494.48). HPLC (purity): 100%. ^1^H NMR (DMSO-*d*_6_): δ = 12.94 (s, 1 H, N*H*), 8.50 (bs, 1 H, C^7^*H*_Bnth_), 7.92 (d, ^3^*J* = 8.5 Hz, 1 H, C^4^*H*_Bnth_), 7.83 (d, ^3^*J* = 6.5 Hz, 1 H, C^6^*H*_Pyr_), 7.75 (dd, ^3^*J* = 8.6 Hz, ^4^*J* = 1.6 Hz, 1 H, C^5^*H*_Bnth_), 7.41–7.35 (m, 1 H, C^4^*H*_Benz_), 7.30–7.25 (m, 1 H, C^3^*H*_Benz_), 7.19 (td, ^3^*J* = 7.5 Hz, ^4^*J* = 1.1 Hz, C^5^*H*_Benz_), 7.06–7.02 (m, 1 H, C^6^*H*_Benz_), 6.28 (d, ^3^*J* = 6.5 Hz, 1 H, C^5^*H*_Pyr_), 5.31 (s, 2 H, N-C*H*_2_), 4.33 (s, 2 H, C*H*_2_-C=O) ppm. ^13^C NMR (DMSO-*d*_6_): δ = 167.2 (CH_2_-*C*=O), 162.1 (*C*^2^_Pyr_), 161.0 (*C*^2^_Bnth_), 160.8 (*C*^4^_Pyr_), 159.9 (d, ^1^*J*_CF_ = 245.1 Hz, C^2^_Benz_), 152.3 (*C*^6^H_Pyr_), 151.3 (*C*^3a^_Bnth_), 132.0 (*C*^7a^_Bnth_), 129.7 (d, ^3^*J*_CF_ = 8.2 Hz, *C*^4^_Benz_), 127.5 (d, ^3^*J*_CF_ = 3.5 Hz, *C*^6^_Benz_), 124.9 (d, ^4^*J*_CF_ = 3.4 Hz, *C*^5^_Benz_), 124.6 (d, ^1^*J*_CF_ = 271.8 Hz, CF_3_), 123.9 (d, ^2^*J*_CF_ = 31.5 Hz, C^6^_Bnth_), 123.0 (d, ^3^*J*_CF_ = 3.5 Hz, *C*^5^_Bnth_), 121.8 (d, ^2^*J*_CF_ = 14.0 Hz, *C*^1^_Benz_), 121.1 (*C*^4^_Bnth_), 120.0 (d, ^3^*J*_CF_ = 4.1 Hz, *C*^7^_Bnth_), 115.6 (d, ^2^*J*_CF_ = 21.0 Hz, *C*^3^_Benz_), 110.2 (*C*^5^_Pyr_), 41.0 (d, ^3^*J*_CF_ = 5.0 Hz, N-*C*H_2_), 35.8 (*C*H_2_-C=O) ppm. MS (ESI, 70 eV) *m*/*z* = 276.9 [C_13_H_10_FN_2_O_2_S]^+^. IR: $\tilde{v}$ = 3142, 3048, 2968, 2916, 1647, 1555, 1491, 1377, 1155, 1115, 1084, 826, 756, 679, cm^−1^.

### 4.10. Molecular Modeling

Molecular modeling was performed on a DELL Precision T5500 8-core system. For visualization Maestro version 11.1, Schrödinger LLC, (New York, NY, USA, 2017) was used. Protein crystal structures were prepared prior to docking by the Protein Preparation Wizard using default settings. The X-ray crystal structure refinement process included the addition of hydrogen atoms, optimization of hydrogen bonds, and removal of atomic clashes. Missing side chains and loops were filled in with Prime, and water molecules were deleted. Small molecule ligands were prepared to create energetically minimized 3D geometries as well as tautomer and ionization states using LigPrep. The ligand MgATP was energetically minimized using MacroModel. For ATP-docking, a homology model of CK1δ (PDB: 4twc) was created based on crystal structure PDB 1csn, a binary complex of CK1 with MgATP [23]. Mutations were introduced into the model by using the biologics tool residue scanning and mutation with side-chain prediction and backbone minimization. Receptor grid generation was performed by Glide. For ligand docking, either the Glide XP workflow was used (default settings) or induced fit docking was used (glide setting: XP). For MgATP, induced fit docking automatic side-chain trimming was activated and H-bond constraints to the hinge binder residues Leu-85 and Glu-83 were specified. Energetically minimized small molecule ligand conformations were docked into the apo active site of the protein. Plausible binding poses were determined and subsequently ranked based on their calculated docking score.

### 4.11. Cell Lines and Transfection

Parental HeLa cells, as well as HeLa *CSNK1D* knock-out cells (CK1δ^−/−^) (generated by CRISP/Cas9 technology and provided by EDIGENE, Beijing, China), were cultured in Dulbecco’s Modified Eagle medium (DMEM). HCT-116 and HT-29 colon cancer cells were cultured in McCoy’s 5A medium whereas SW480 and SW620 colon cancer cells were cultured in Leibovitz’s L-15 medium. All media were supplemented with 10% (*v*/*v*) fetal bovine serum (FBS), 100 units/mL penicillin, and 100 µg/mL streptomycin. DMEM-based medium for the culture of HeLa *CSNK1D* knock-out cells was additionally supplemented with 1 μg/mL puromycin. Cell cultures were maintained at 37 °C in a humidified atmosphere containing 5% CO_2_. Cells were transfected using Effectene transfection reagent (Qiagen, Hilden, Germany) according to the manufacturer’s instructions. For selection of stably transfected clones, transfected cells were grown in selective medium, containing 0.6 mg/mL of G-418 (Calbiochem-Merck Millipore, Darmstadt, Germany).

### 4.12. MTT Viability Assay

For performing MTT viability assays, cells were seeded in a 96-well plate (5 × 10^4^ cells/well for HeLa cells (parental, CK1δ^−/−^, or CK1δ^−/−^ cells stably transfected with wild type or mutant CK1δ), HCT-116, and HT-29 cells or 7.5 × 10^4^ cell/well for SW480 and SW620 cells). After 24 h, old medium was replaced with fresh medium containing either the small molecule of interest or DMSO as control. After 48 h of treatment, 10 μL MTT (5 mg/mL) was added and incubated at 37 °C for 4 h. The medium was then gently removed and 100 μL of acidic isopropanol was added (90% (*v*/*v*) isopropanol, 10% (*v*/*v*) 1 N HCl). Cell culture plates were then shaken for 30 min at room temperature. Absorbance was measured at 570 nm with TriStar^2^ LB942 Microplate Reader (Berthold Technologies, Bad Wildbad, Germany) and data analysis was performed using GraphPad Prism 6 (GraphPad Software, San Diego, CA, USA).

### 4.13. Statistical Methods

Results were presented as the mean of experiments at least performed in triplicate ± SD. In the case of IC_50_ data, the standard error was calculated based on values obtained from three independent experiments. Enzyme kinetic data as well as IC_50_ and EC_50_ data was calculated using GraphPad Prism 6 (GraphPad Software, San Diego, CA, USA). Other data calculation and presentation were performed using either GraphPad Prism 6 (GraphPad Software, San Diego, CA, USA) or Microsoft Excel 2016 (Microsoft, Redmond, Washington, USA). Where indicated, statistical analysis was done by performing two-way ANOVA. *P*-values smaller than 0.05 were considered to be statistically significant. Levels of significance were indicated by asterisks as follows: * *p* ≤ 0.05; ** *p* ≤ 0.01; *** *p* ≤ 0.001; **** *p* ≤ 0.0001; ns, not significant.

## 5. Conclusions

In order to develop treatment strategies for application in personalized medicine, profound understanding of the effects of mutations of CK1δ, being associated with increased oncogenic potential, is crucial. In the present study, we characterized several mutations of CK1δ, which resulted in an increase of kinase activity. Furthermore, we demonstrated conclusively that CK1-specific small molecule inhibitors also have the potential to inhibit the tested CK1δ mutants. By selectively targeting tumorigenesis-associated CK1δ mutants instead of wild type CK1δ, tumor-specific effects could be obtained, offering a much wider therapeutic window than for targeting CK1δ in general. In this regard, new CK1 isoform- and mutant-specific inhibitors like the ones described in the present study represent interesting tools for future therapeutic applications in personalized medicine.

## Figures and Tables

**Table 1:** List of selected mutations in *CSNK1D* and the respective cancer types.

Mutation	Cancer Type
L25P	stomach adenocarcinoma, lung adenocarcinoma
A36V	colorectal adenocarcinoma, esophageal squamous cell carcinoma, mixed cancer types
R115H	colorectal adenocarcinoma, head and neck squamous cell carcinoma, uterine endometrioid carcinoma, mixed cancer types
R127L	lung squamous cell carcinoma
R127Q	bladder urothelial carcinoma
I148M	cutaneous melanoma
R160H	colorectal adenocarcinoma, mixed cancer types
R160P	colon adenocarcinoma
R160S	mixed cancer types
R168H	uterine endometrioid carcinoma, mixed cancer types
R178W	colorectal adenocarcinoma, mixed cancer types
E247K	uterine endometrioid carcinoma, rectal adenocarcinoma
L252P	mixed cancer types
R299Q	mixed cancer types
R316G	mixed cancer types
Q399 *	pancreatic adenocarcinoma
H414Y	pancreatic adenocarcinoma

Mutations of *CSNK1D* selected for further analysis after performing a database query at cBioPortal for Cancer Genomics are listed together with information about the type(s) of cancer in which the respective mutations have been identified [5,6]. Abbreviations: A, alanine; E, glutamic acid; G, glycine; H, histidine; I, isoleucine; K, lysine; L, leucine; M, methionine; P, proline; Q, glutamine; R, arginine; S, serine; V, valine; W, tryptophan; Y, tyrosine; *, stop codon.

**Table 2:** Residual activity of CK1δ wild type or mutants after treatment with CK1-specific inhibitors.

CK1δ Variant	Bischof-5	Richter-2	IWP-2	IWP-4
WT	50 ± 17	50 ± 15	50 ± 6	50 ± 6
A36V	**43 ± 15**	109 ± 33	55 ± 6	**49 ± 7**
R115H	**36 ± 10**	211 ± 79	71 ± 34	**46 ± 5**
R127L	50 ± 11	88 ± 34	**42 ± 9**	**48 ± 10**
R127Q	**24 ± 8**	**19 ± 2**	**37 ± 5**	**36 ± 11**
R168H	**30 ± 16**	**31 ± 16**	50 ± 9	61 ± 19
R299Q	64 ± 22	69 ± 17	**36 ± 20**	91 ± 60
Q399*	75 ± 30	50 ± 14	53 ± 27	96 ± 79

Kinase activity of CK1δ^WT^ and mutants was tested in presence of the CK1-specific inhibitors Bischof-5, Richter-2, IWP-2, and IWP-4, which were used in the IC_50_ concentration previously determined for CK1δ^WT^ [14,15,17]. Residual activity is presented as normalized values using the respective DMSO control as a reference (100%). Values were adjusted in order to obtain half-maximal inhibition values for CK1δ^WT^ (IC_50_; 50%). Data is represented as mean values ± SD of normalized triplicates. Residual kinase activities lower than 50% are highlighted in bold. Abbreviations: A, alanine; V, valine; R, arginine; H, histidine; L, leucine; Q, glutamine; WT, wild type; *, stop codon.

## Supplementary Materials

Supplementary Materials can be found at https://www.mdpi.com/1422-0067/20/24/6184/s1.

## Abbreviations

*	Stop codon
A	Alanine
aa	Amino acid
CLL	Chronic lymphocytic leukemia
CPM	Counts per minute
CRC	Colon and rectal cancer
DMEM	Dulbecco’s Modified Eagle Medium
DMSO	Dimethyl sulfoxide
E, Glu	Glutamic acid
EC_50_	50% Effective concentration
F	Phenylalanine
fwd	Forward
G	Glycine
GST	Glutathione S-transferase
H, His	Histidine
I, Ile	Isoleucine
IC_50_	50% inhibitory concentration
IWP	Inhibitor of Wnt production
K, Lys	Lysine
k_cat_	Catalytic constant/turnover number
K_M_	Michaelis constant
L, Leu	Leucine
M	Methionine
ns	Not significant
P	Proline
PDB	Protein data bank
Q	Glutamine
R	Arginine
rev	Reverse
RT	Room temperature
S	Serine
SD	Standard deviation
T	Threonine
TV	Transcription variant
UICC	Union Internationale Contre le Cancer
V	Valine
V_max_	Maximal enzyme reaction velocity
W	Tryptophan
WT	Wild type
Y	Tyrosine

## References

[1] Kumar A., Rajendran V., Sethumadhavan R., Purohit R. (2014). Relationship between a point mutation S97C in CK1delta protein and its affect on ATP-binding affinity. J. Biomol. Struct. Dyn..

[2] Richter J., Ullah K., Xu P., Alscher V., Blatz A., Peifer C., Halekotte J., Leban J., Vitt D., Holzmann K. (2015). Effects of altered expression and activity levels of CK1delta and varepsilon on tumor growth and survival of colorectal cancer patients. Int. J. Cancer.

[3] Tsai I.C., Woolf M., Neklason D.W., Branford W.W., Yost H.J., Burt R.W., Virshup D.M. (2007). Disease-associated casein kinase I delta mutation may promote adenomatous polyps formation via a Wnt/beta-catenin independent mechanism. Int. J. Cancer.

[4] Hirner H., Gunes C., Bischof J., Wolff S., Grothey A., Kuhl M., Oswald F., Wegwitz F., Bosl M.R., Trauzold A. (2012). Impaired CK1 delta activity attenuates SV40-induced cellular transformation in vitro and mouse mammary carcinogenesis in vivo. PLoS ONE.

[5] Cerami E., Gao J., Dogrusoz U., Gross B.E., Sumer S.O., Aksoy B.A., Jacobsen A., Byrne C.J., Heuer M.L., Larsson E. (2012). The cBio cancer genomics portal: An open platform for exploring multidimensional cancer genomics data. Cancer Discov..

[6] Gao J., Aksoy B.A., Dogrusoz U., Dresdner G., Gross B., Sumer S.O., Sun Y., Jacobsen A., Sinha R., Larsson E. (2013). Integrative analysis of complex cancer genomics and clinical profiles using the cBioPortal. Sci. Signal..

[7] Brockschmidt C., Hirner H., Huber N., Eismann T., Hillenbrand A., Giamas G., Radunsky B., Ammerpohl O., Bohm B., Henne-Bruns D. (2008). Anti-apoptotic and growth-stimulatory functions of CK1 delta and epsilon in ductal adenocarcinoma of the pancreas are inhibited by IC261 in vitro and in vivo. Gut.

[8] Toyoshima M., Howie H.L., Imakura M., Walsh R.M., Annis J.E., Chang A.N., Frazier J., Chau B.N., Loboda A., Linsley P.S. (2012). Functional genomics identifies therapeutic targets for MYC-driven cancer. Proc. Natl. Acad. Sci. USA.

[9] Cheong J.K., Nguyen T.H., Wang H., Tan P., Voorhoeve P.M., Lee S.H., Virshup D.M. (2011). IC261 induces cell cycle arrest and apoptosis of human cancer cells via CK1delta/varepsilon and Wnt/beta-catenin independent inhibition of mitotic spindle formation. Oncogene.

[10] Fohr K.J., Knippschild U., Herkommer A., Fauler M., Peifer C., Georgieff M., Adolph O. (2017). State-dependent block of voltage-gated sodium channels by the casein-kinase 1 inhibitor IC261. Investig. New Drugs.

[11] Janovska P., Verner J., Kohoutek J., Bryjova L., Gregorova M., Dzimkova M., Skabrahova H., Radaszkiewicz T., Ovesna P., Vondalova Blanarova O. (2018). Casein kinase 1 is a therapeutic target in chronic lymphocytic leukemia. Blood.

[12] Walton K.M., Fisher K., Rubitski D., Marconi M., Meng Q.J., Sladek M., Adams J., Bass M., Chandrasekaran R., Butler T. (2009). Selective inhibition of casein kinase 1 epsilon minimally alters circadian clock period. J. Pharmacol. Exp. Ther..

[13] Bibian M., Rahaim R.J., Choi J.Y., Noguchi Y., Schurer S., Chen W., Nakanishi S., Licht K., Rosenberg L.H., Li L. (2013). Development of highly selective casein kinase 1delta/1epsilon (CK1delta/epsilon) inhibitors with potent antiproliferative properties. Bioorg. Med. Chem. Lett..

[14] Bischof J., Leban J., Zaja M., Grothey A., Radunsky B., Othersen O., Strobl S., Vitt D., Knippschild U. (2012). 2-Benzamido-N-(1H-benzo[d]imidazol-2-yl)thiazole-4-carboxamide derivatives as potent inhibitors of CK1delta/epsilon. Amino Acids.

[15] Garcia-Reyes B., Witt L., Jansen B., Karasu E., Gehring T., Leban J., Henne-Bruns D., Pichlo C., Brunstein E., Baumann U. (2018). Discovery of Inhibitor of Wnt Production 2 (IWP-2) and Related Compounds As Selective ATP-Competitive Inhibitors of Casein Kinase 1 (CK1) delta/epsilon. J. Med. Chem..

[16] Halekotte J., Witt L., Ianes C., Kruger M., Buhrmann M., Rauh D., Pichlo C., Brunstein E., Luxenburger A., Baumann U. (2017). Optimized 4,5-Diarylimidazoles as Potent/Selective Inhibitors of Protein Kinase CK1delta and Their Structural Relation to p38alpha MAPK. Molecules.

[17] Richter J., Bischof J., Zaja M., Kohlhof H., Othersen O., Vitt D., Alscher V., Pospiech I., Garcia-Reyes B., Berg S. (2014). Difluoro-dioxolo-benzoimidazol-benzamides as potent inhibitors of CK1delta and epsilon with nanomolar inhibitory activity on cancer cell proliferation. J. Med. Chem..

[18] Miller K.D., Nogueira L., Mariotto A.B., Rowland J.H., Yabroff K.R., Alfano C.M., Jemal A., Kramer J.L., Siegel R.L. (2019). Cancer treatment and survivorship statistics, 2019. Cancer J. Clin..

[19] Guinney J., Dienstmann R., Wang X., de Reynies A., Schlicker A., Soneson C., Marisa L., Roepman P., Nyamundanda G., Angelino P. (2015). The consensus molecular subtypes of colorectal cancer. Nature Med..

[20] Paschke S., Jafarov S., Staib L., Kreuser E.D., Maulbecker-Armstrong C., Roitman M., Holm T., Harris C.C., Link K.H., Kornmann M. (2018). Are Colon and Rectal Cancer Two Different Tumor Entities? A Proposal to Abandon the Term Colorectal Cancer. Int. J. Mol. Sci..

[21] Bischof J., Randoll S.J., Sussner N., Henne-Bruns D., Pinna L.A., Knippschild U. (2013). CK1delta kinase activity is modulated by Chk1-mediated phosphorylation. PLoS ONE.

[22] Chen B., Dodge M.E., Tang W., Lu J., Ma Z., Fan C.W., Wei S., Hao W., Kilgore J., Williams N.S. (2009). Small molecule-mediated disruption of Wnt-dependent signaling in tissue regeneration and cancer. Nature Chem. Biol..

[23] Xu R.M., Carmel G., Sweet R.M., Kuret J., Cheng X. (1995). Crystal structure of casein kinase-1, a phosphate-directed protein kinase. EMBO J..

[24] Berman H.M., Westbrook J., Feng Z., Gilliland G., Bhat T.N., Weissig H., Shindyalov I.N., Bourne P.E. (2000). The Protein Data Bank. Nucleic Acids Res..

[25] Liu C.Y., Li Y., Ding J.Y., Dong D.W., Han F.S. (2014). The development of copper-catalyzed aerobic oxidative coupling of H-tetrazoles with boronic acids and an insight into the reaction mechanism. Chemistry.

[26] Abd El Kader K.F., El Bialy S.A.A., El-Ashmawy M.B., Boykin D.W. (2012). Pirfenidone structural isosteres: Design, synthesis and spectral study. Heterocycl. Commun..

[27] Roopan S.M., Khan F.R.N., Mandal B.K. (2010). Fe nano particles mediated C–N bond-forming reaction: Regioselective synthesis of 3-[(2-chloroquinolin-3-yl)methyl]pyrimidin-4(3H)ones. Tetrahedron. Lett..

[28] Xu J., Yadan J.-C. (1995). A new and convenient synthesis of imidazole-2-thiones from imidazoles. Synlett.

[29] Nekkaa I., Palko M., Mandity I.M., Fulop F. (2018). Continuous-flow retro-Diels-Alder reaction: An efficient method for the preparation of pyrimidinone derivatives. Beilstein J. Org. Chem..

[30] Wang X., Moon J., Dodge M.E., Pan X., Zhang L., Hanson J.M., Tuladhar R., Ma Z., Shi H., Williams N.S. (2013). The development of highly potent inhibitors for porcupine. J. Med. Chem..

[31] Milne D.M., Looby P., Meek D.W. (2001). Catalytic activity of protein kinase CK1 delta (casein kinase 1delta) is essential for its normal subcellular localization. Exp. Cell Res..

[32] Rivers A., Gietzen K.F., Vielhaber E., Virshup D.M. (1998). Regulation of casein kinase I epsilon and casein kinase I delta by an in vivo futile phosphorylation cycle. J. Biol. Chem..

[33] Xu P., Ianes C., Gartner F., Liu C., Burster T., Bakulev V., Rachidi N., Knippschild U., Bischof J. (2019). Structure, regulation, and (patho-)physiological functions of the stress-induced protein kinase CK1 delta (CSNK1D). Gene.

[34] Giamas G., Hirner H., Shoshiashvili L., Grothey A., Gessert S., Kuhl M., Henne-Bruns D., Vorgias C.E., Knippschild U. (2007). Phosphorylation of CK1delta: Identification of Ser370 as the major phosphorylation site targeted by PKA in vitro and in vivo. Biochem. J..

[35] Ianes C., Xu P., Werz N., Meng Z., Henne-Bruns D., Bischof J., Knippschild U. (2016). CK1delta activity is modulated by CDK2/E- and CDK5/p35-mediated phosphorylation. Amino Acids.

[36] Meng Z., Bischof J., Ianes C., Henne-Bruns D., Xu P., Knippschild U. (2016). CK1delta kinase activity is modulated by protein kinase C alpha (PKCalpha)-mediated site-specific phosphorylation. Amino Acids.

[37] Melcher R., Steinlein C., Feichtinger W., Muller C.R., Menzel T., Luhrs H., Scheppach W., Schmid M. (2000). Spectral karyotyping of the human colon cancer cell lines SW480 and SW620. Cytogenet. Cell Genet..

[38] Schehr M., Ianes C., Weisner J., Heintze L., Muller M.P., Pichlo C., Charl J., Brunstein E., Ewert J., Lehr M. (2019). 2-Azo-, 2-diazocine-thiazols and 2-azo-imidazoles as photoswitchable kinase inhibitors: Limitations and pitfalls of the photoswitchable inhibitor approach. Photochem. Photobiol. Sci..

