# Supplementary material for: Newly Developed CK1-Specific Inhibitors Show Specifically Stronger Effects on CK1 Mutants and Colon Cancer Cell Lines
Source: Int J Mol Sci. 2019 Dec 7;20(24):6184. doi: 10.3390/ijms20246184 (PMC6941124; doi:10.3390/ijms20246184)
Supplement: Supplementary file 1 [file ijms-20-06184-s001.zip › Supplementary Table 2.pdf]

Supplementary Table 2. Primers used for site-directed mutagenesis. Sequences of primers used for site-directed mutagenesis of pGEX6-P3 or pcDNA3.1 expression vectors, coding for GST-CK1δ and His-CK1δ, respectively. Abbreviations: fwd, forward; rev, reverse.

mutant	nucleotide exchange	primer	primer sequence
A36V	GCC-GTC	fwd	5’-gcaggagaagaggttgtcatcaagcttgaatgtg-3’
A36V	GCC-GTC	rev	5’-cacattcaagcttgatgacaacctcttctcctgca-3’
L25V	CTC-CCC	fwd	5’-cttcggagacatctatcccggtacggacattgctg-3’
L25V	CTC-CCC	rev	5’-cagcaatgtccgtaccgggatagatgtctccgaag-3’
R115H	CGC-CAC	fwd	5’-cttgctgaccaaatgatcagtcacatcgaatacattcattcaaag-3’
R115H	CGC-CAC	rev	5’-ctttgaatgaatgtattcgatgtgactgatcatttggtcagcaag-3’
R127L	CGG-CTG	fwd	5’-aaagaacttcatccacctggatgtgaagccagaca-3’
R127L	CGG-CTG	rev	5’-tgtctggcttcacatccaggtggatgaagttcttt-3’
R127Q	CGG-CAG	fwd	5’-aaagaacttcatccaccaggatgtgaagccagaca-3’
R127Q	CGG-CAG	rev	5’-tgtctggcttcacatcctggtggatgaagttcttt-3’
I148M	ATC-ATG	fwd	5’-ggcaacctggtgtacatcatggacttcgggct-3’
I148M	ATC-ATG	rev	5’-agcccgaagtccatgatgtacaccaggttgcc-3’
R160H	CGC-CAC	fwd	5’-taccgggatgcacacacccaccagcac-3’
R160H	CGC-CAC	rev	5’-gtgctggtgggtgtgtgcatcccggta-3’
R160P	CGC-CCC	fwd	5’-taccgggatgcacccacccaccagcac-3’
R160P	CGC-CCC	rev	5’-gtgctggtgggtgggtgcatcccggta-3’
R160S	CGC-AGC	fwd	5’-taccgggatgcaagcacccaccagcac-3’
R160S	CGC-AGC	rev	5’-gtgctggtgggtgcttgcatcccggta-3’
R168H	CGT-CAT	fwd	5’-ccagcacatcccctatcatgagaacaagaacctca-3’
R168H	CGT-CAT	rev	5’-tgaggttcttgttctcatgataggggatgtgctgg-3’
R178W	CGG-TGG	fwd	5’-cggggacggcgtggtacgcctcc-3’
R178W	CGG-TGG	rev	5’-ggaggcgtaccacgccgtccccg-3’
E247K	GAA-AAA	fwd	5’-gtaaaggctacccttccaaatttgccacatacctg-3’
E247K	GAA-AAA	rev	5’-caggtatgtggcaaatttggaagggtagcctttac-3’
L252P	CTG-CCG	fwd	5’-cgaatttgccacatacccgaatttctgccgttcct-3’
L252P	CTG-CCG	rev	5’-aggaacggcagaaattcgggtatgtggcaaattcg-3’
R299Q	CGG-CAG	fwd	5’-tggtgccagccaggccgccgatg-3’
R299Q	CGG-CAG	rev	5’-catcggcggcctggctggcacca-3’
R316G	AGA-GGA	fwd	5’-gaggagcggctgggacactcgcgga-3’
R316G	AGA-GGA	rev	5’-tccgcgagtgtcccagccgctcctc-3’
Q399*	CAG-TAG	fwd	5’-ctcgcatgtccacctcatagattcctggtcg-3'
Q399*	CAG-TAG	rev	5’-cgaccaggaatctatgaggtggacatgcgag-3'

H414Y	CAC-TAC	fwd	5’-gtcttcagtctgtcgtgtaccgatgaggatccaag-3’
H414Y	CAC-TAC	rev	5’-cttggatcctcatcggtacacgacagactgaagac-3’
