# Supplementary material for: Newly Developed CK1-Specific Inhibitors Show Specifically Stronger Effects on CK1 Mutants and Colon Cancer Cell Lines
Source: Int J Mol Sci. 2019 Dec 7;20(24):6184. doi: 10.3390/ijms20246184 (PMC6941124; doi:10.3390/ijms20246184)
Supplement: Supplementary file 1 [file ijms-20-06184-s001.zip › Supplementary Table 1.pdf]

Supplementary Table 1. Enzyme kinetic parameters (V_{max}, K_M, k_{cat}, k_{cat}/K_M) of CK1δ wild type and mutants for three different substrates. Kinetic parameters were determined for CK1δ wild type and mutants using either α-casein, GST-β-catenin$^{1-181}$, or GST-p53$^{1-64}$ as substrate. Data is presented as mean values ± standard deviation (SD) for experiments performed in triplicate. Abbreviations: A, alanine; E, glutamic acid; G, glycine; GST, glutathione S-transferase; H, histidine; I, isoleucine; K, lysine; k_{cat}, turn-over number; L, leucine; M, methionine; min, minute; µM, micromol; P, proline; pmol, picomol; Q, glutamine; R, arginine; S, serine; sec, second; V, valine; V_{max}, maximum enzyme reaction velocity; W, tryptophan; WT, wild type; Y, tyrosine; *, stop codon.

**CK1δ mutant**	**α-casein**				**GST-β-catenin$^{1-181}$**				**GST-β-p53$^{1-64}$**			
	**V_{max} [pmol/min/mg]**	**K_M [µM]**	**k_{cat} [sec^{-1}]**	**k_{cat}/K_M [sec^{-1} μM^{-1}]**	**V_{max} [pmol/min/mg]**	**K_M [µM]**	**k_{cat} [sec^{-1}]**	**k_{cat}/K_M [sec^{-1} μM^{-1}]**	**V_{max} [pmol/min/mg]**	**K_M [µM]**	**k_{cat} [sec^{-1}]**	**k_{cat}/K_M [sec^{-1} μM^{-1}]**
**WT**	336382 ± 24923	11.7 ± 1.8	6051 ± 1569	482 ± 121	5860 ± 819	0.50 ± 0.17	100 ± 8	197 ± 56	4173 ± 1238	2.25 ± 1.05	49 ± 12	37.8 ± 6.7
**L25P**	5113 ± 300	2.2 ± 0.5	86 ± 7	40 ± 13	580 ± 74	0.01 ± 0.03	10 ± 3	620 ± 620	464 ± 32	0.09 ± 0.04	8 ± 2	204 ± 59
**A36V**	814548 ± 163379	12.9 ± 5.2	14772 ± 4565	1040 ± 346	14993 ± 2258	0.36 ± 0.15	251 ± 14	715 ± 192	2267 ± 327	0.54 ± 0.22	38 ± 9	72 ± 6
**R115H**	62918 ± 7754	5.5 ± 1.9	1049 ± 156	193 ± 6	2145 ± 352	0.46 ± 0.19	30 ± 9	145 ± 115	13379 ± 2539	0.65 ± 0.31	236 ± 43	333 ± 120
**R127L**	942637 ± 614292	73 ± 59	10706 ± 3621	220 ± 35	48345 ± 13700	0.46 ± 0.33	762 ± 22	2744 ± 1333	12439 ± 5139	1.37 ± 1.06	236 ± 145	281 ± 284
**R127Q**	1184000 ± 329292	65 ± 23	24638 ± 11890	306 ± 42	42840 ± 7692	0.61 ± 0.25	1029 ± 784	1527 ± 1063	130054 ± 15720	0.17 ± 0.09	2212 ± 112	8916 ± 1403
**I148M**	85476 ± 11630	16 ± 4	1430 ± 193	92 ± 11	267 ± 41	0.24 ± 0.13	6 ± 4	25 ± 17	387 ± 87	0.48 ± 0.31	14 ± 17	17 ± 13
**R160H**	132275 ± 24227	10 ± 4	1593 ± 391	254 ± 120	5227 ± 978	0.81 ± 0.30	91 ± 20	111 ± 30	414 ± 114	0.70 ± 0.47	8 ± 3	10 ± 4
**R160P**	156772 ± 16423	6.1 ± 1.7	2722 ± 650	436 ± 119	511 ± 71	0.43 ± 0.16	9 ± 2	20 ± 7	1754 ± 263	0.48 ± 0.20	30 ± 10	74 ± 35
**R160S**	174988 ± 27722	15 ± 5	2361 ± 579	201 ± 83	591 ± 90	0.47 ± 0.18	11 ± 2	22 ± 10	601 ± 182	1.05 ± 0.66	11 ± 4	10 ± 4
**R168H**	488699 ± 130163	36 ± 14	5043 ± 255	279 ± 3	4769 ± 334	0.10 ± 0.03	81 ± 14	1543 ± 255	5510 ± 2	0.87 ± 0.01	101 ± 36	116 ± 47
**R178W**	19880 ± 1651	9.7 ± 1.8	336 ± 27	34 ± 4	2397 ± 212	0.19 ± 0.06	42 ± 10	226 ± 98	1336 ± 193	1.30 ± 0.36	23 ± 6	17 ± 4
**E247K**	243 ± 30	1.8 ± 1.0	6 ± 2	0.6 ± 0.3	74 ± 12	0.01 ± 0.03	1.3 ± 0.4	110 ± 69	98 ± 4	0.01 ± 0.01	1.5 ± 0.1	278 ± 22
**L252P**	195 ± 37	1.1 ± 1.0	3.3 ± 0.2	3 ± 1	46 ± 3	0.01 ± 0.01	0.8 ± 0.1	143 ± 15	34 ± 2	0.01 ± 0.01	0.6 ± 0.1	29 ± 9
**R299Q**	705191 ± 127744	33 ± 9	13270 ± 5087	364 ± 98	9545 ± 4369	1.84 ± 1.27	123 ± 43	127 ± 44	4471 ± 1157	2.45 ± 0.97	79 ±22	31 ± 5
**R316G**	191294 ± 20894	21 ± 4	3208 ± 263	155 ± 22	2527 ± 740	1.57 ± 0.73	44 ± 15	28 ± 6	880 ± 192	0.74 ± 0.39	15 ± 3	20 ± 1
**Q399***	413321 ± 104175	35 ± 13	7190 ± 2191	197 ± 20	3701 ± 1412	1.20 ± 0.79	46 ± 18	61 ± 19	2250 ± 1442	3.17 ± 2.86	25 ± 1	17 ± 1
**H414Y**	110421 ± 33216	26 ± 12	1999 ± 459	73 ± 25	1525 ± 411	1.74 ± 0.72	26 ± 5	15 ± 2	410 ± 83	0.68 ± 0.34	7 ± 1	10 ± 2
