# Supplementary figures and images for: Newly Developed CK1-Specific Inhibitors Show Specifically Stronger Effects on CK1 Mutants and Colon Cancer Cell Lines
Source: Int J Mol Sci. 2019 Dec 7;20(24):6184. doi: 10.3390/ijms20246184 (PMC6941124; doi:10.3390/ijms20246184)

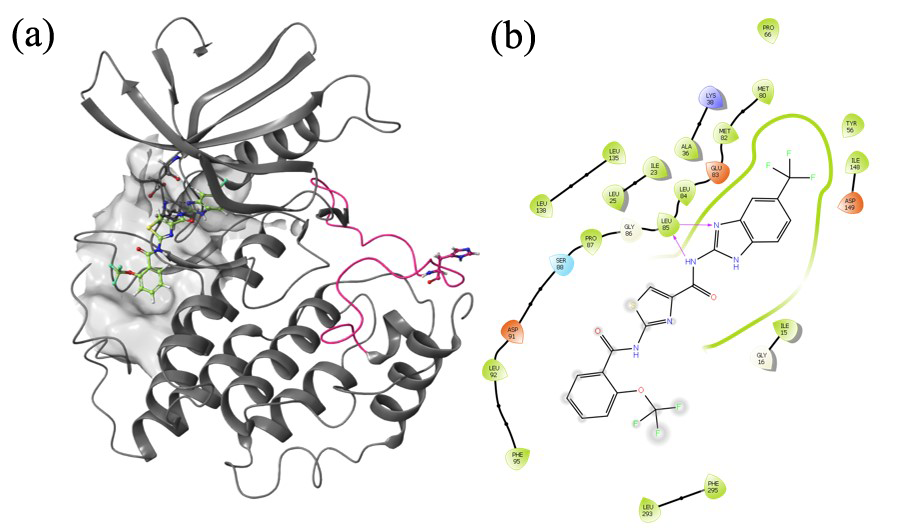

Supplement: Supplementary file 1 [file ijms-20-06184-s001.zip › Supplementary Figure 1.tif]

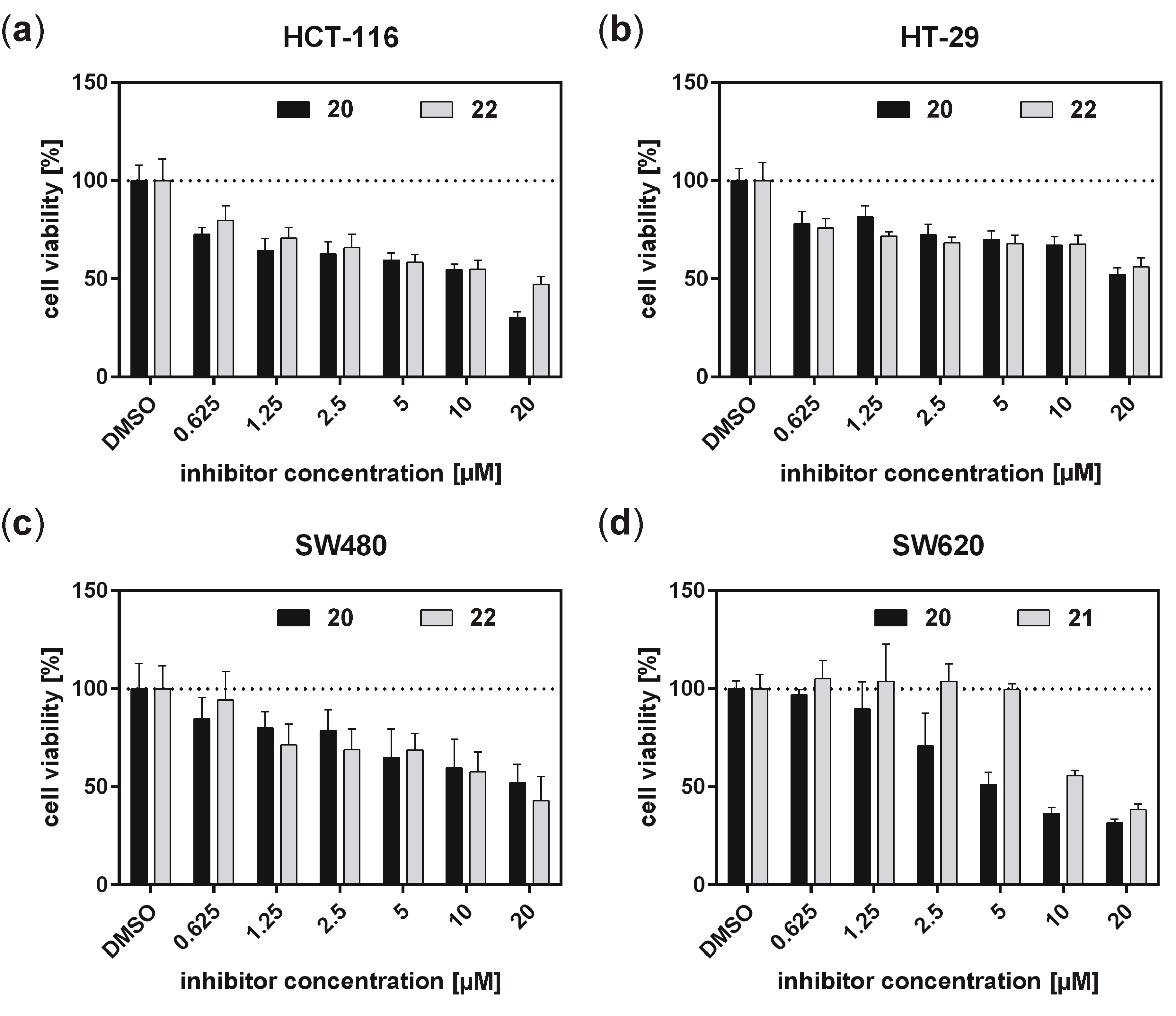

Supplement: Supplementary file 1 [file ijms-20-06184-s001.zip › Supplementary Figure 2.tif]
